# Vapor-induced miscibility switching and optical response in a functional molecular liquid–pillar[5]arene system

**DOI:** 10.1039/d6sc03713e

**Published:** 2026-07-21

**Authors:** Yosuke Tani, Keisuke Wada, Yuya Oshima, Takanori Nakane, Akihiro Kawamoto, Genji Kurisu, Takashi Tachikawa, Shunsuke Ohtani, Kenichi Kato, Tomoki Ogoshi

**Affiliations:** a Department of Chemistry, Graduate School of Science, The University of Osaka Toyonaka Osaka 560-0043 Japan; b Institute of Transformative Bio-Molecules (ITbM), Nagoya University Furo, Chikusa Nagoya 464-8601 Japan tani.yosuke.y1@f.mail.nagoya-u.ac.jp; c Department of Synthetic Chemistry and Biological Chemistry, Graduate School of Engineering, Kyoto University Katsura, Nishikyo-ku Kyoto 615-8510 Japan ogoshi@sbchem.kyoto-u.ac.jp; d Institute for Protein Research, The University of Osaka 3-2 Yamadaoka Suita Osaka 565-0871 Japan gkurisu@protein.osaka-u.ac.jp; e JEOL YOKOGUSHI Research Alliance Laboratories, Graduate School of Frontier Biosciences, The University of Osaka 1-3 Yamadaoka Suita Osaka 565-0871 Japan; f Center for Life Photonic Innovation, Kobe University 1-1 Rokkodai-cho, Nada-ku Kobe 657-8501 Japan tachikawa@port.kobe-u.ac.jp; g Department of Chemistry, Graduate School of Science, Kobe University 1-1 Rokkodai-cho, Nada-ku Kobe 657-8501 Japan; h WPI Nano Life Science Institute (WPI-Nano LSI), Kanazawa University Kakuma-machi Kanazawa 920-1192 Japan

## Abstract

Stimulus-responsive control of intermolecular interactions in multicomponent systems is a grand challenge in supramolecular chemistry and materials science. Herein, we demonstrate a stimulus-responsive miscibility switching in a system composed of a functional molecular liquid (FML) and a pillar[5]arene (P5A). Our strategy exploits alkyl chains, which are ubiquitously incorporated in FMLs as a liquifying group, to control the miscibility. P5A should be an ideal counterpart because it is known to encapsulate a linear alkane even in the solid state. An FML bearing two alkyl chains spontaneously forms a complex with P5A, resulting in solidification, accompanied by drastic chromism from yellow to red due to charge-transfer interactions. Moreover, while the FML exhibits room-temperature phosphorescence (RTP) in its liquid state, the emission is quenched upon complexation. Remarkably, exposing the solid to linear alkane vapor triggers the reversal: the red color fades, and RTP is turned on. The real-time microscopic observation reveals the abrupt movement and reshaping of crystals, accompanied by liquid seepage, indicating a vapor-induced crystal transition and concomitant solid–liquid phase separation. This reversible phase mixing/separation process, controlled by competitive host–guest chemistry of cyclic host and FMLs, represents a promising approach for designing stimulus-responsive multicomponent systems with switchable optical and physical properties.

## Abstract 2

Host–guest chemistry of an alkylated molecular liquid with a cyclic host realized a vapor-induced miscibility switching system, exhibiting macroscopic solid–liquid phase separation and turn-on room-temperature phosphorescence.

## Graphical abstract

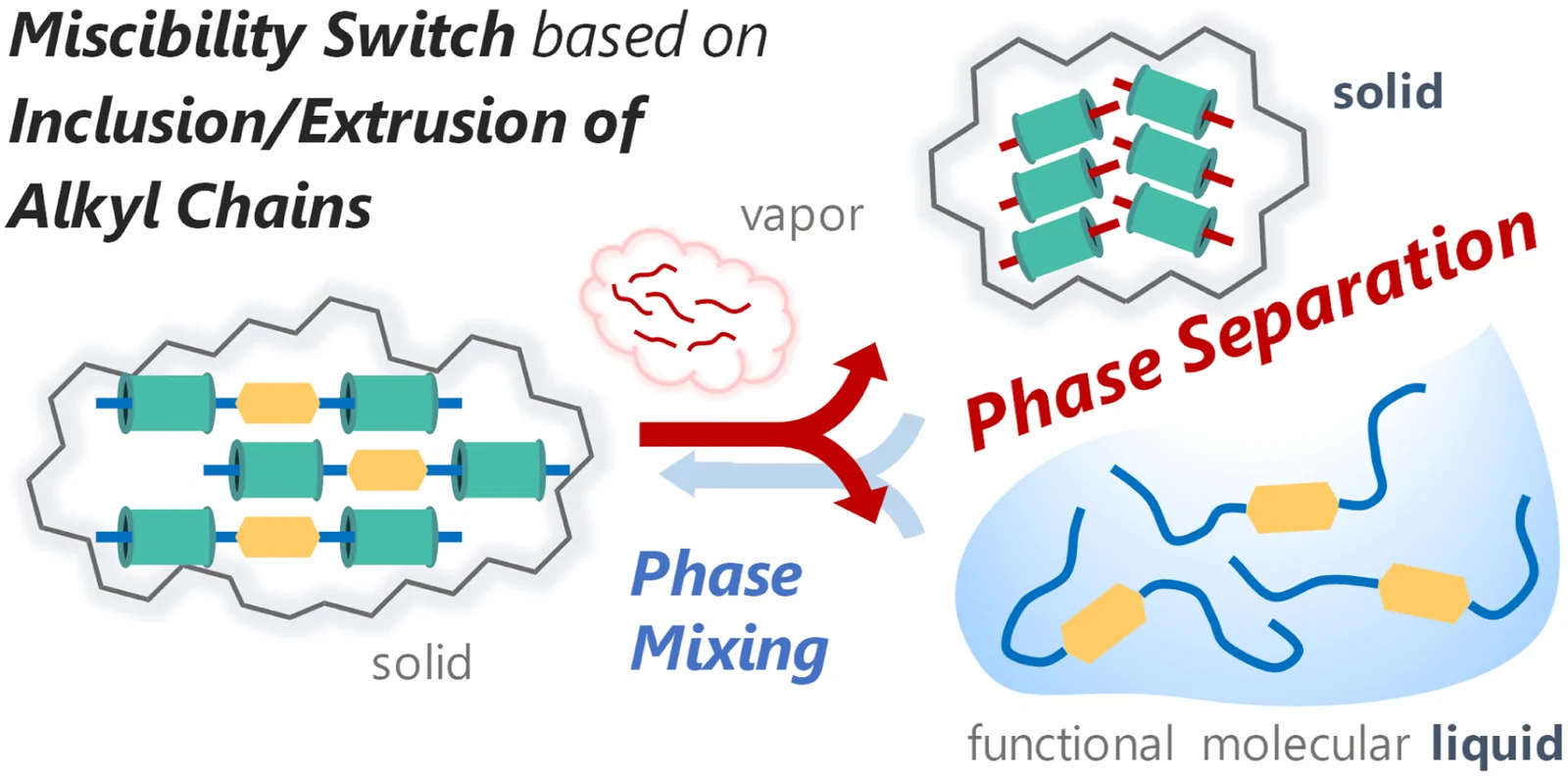

## Introduction

Controlling multicomponent molecular systems and their functions, from a molecular level to a macroscopic scale, is a central issue in supramolecular chemistry and materials science. In particular, stimulus-responsive control of macroscopic miscibility holds great promise. Decreasing miscibility leads to phase separation, which spatially segregates each molecular component, altering the intermolecular interactions such as donor–acceptor interactions, thereby drastically changing its function. Phase separation is pivotal in biological systems, including order–disorder phase separation of lipid bilayer membranes^1^ and liquid–liquid phase separation to form membrane-less organelles.^2^ However, when the multicomponent systems are composed of solid materials, their miscibility at molecular and bulk scales, as well as molecular motions and transportation in the solid states, are limited and hard to predict or design.^3^

In this regard, functional molecular liquids (FMLs) would serve as a promising basis for stimulus-responsive miscibility-switching systems, because FMLs are fluid, non-volatile, and can act as solvents.^4^ Indeed, FML-based multicomponent systems have been actively investigated, whose properties were distinct from the parent FML itself.^5,6^ However, the stimulus-responsive control of miscibility, *i.e.*, reversible phase mixing/phase separation, has never been realized in FML-based multicomponent systems.

**Fig. 1:**
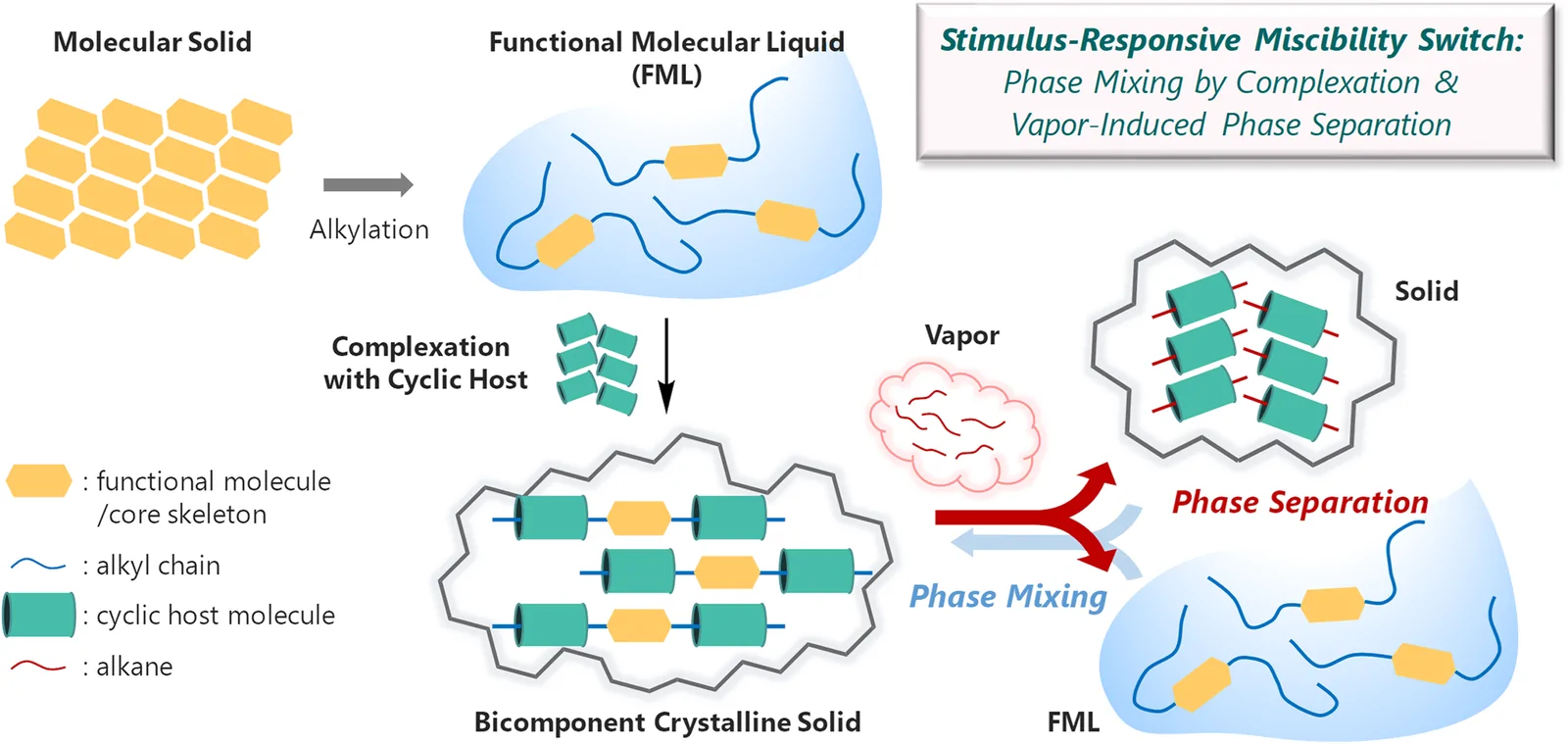
Concept of this work featuring a stimulus-responsive miscibility switch, *i.e.*, reversible phase mixing–phase separation, based on an FML–P*n*A system.

To propose a new design for stimulus-responsive miscibility-switching systems, we focused on alkyl chains, which are ubiquitously found in FMLs (Fig. 1). Introducing alkyl chains to functional core skeletons is a prevailing design of FMLs;^4^ a range of functional π-conjugated materials were liquefied with this design, including fullerene,^7^ porphyrin,^8^ pyrene,^9^ anthracene,^6,10^ carbazole,^11^ naphthalimide,^12^ azobenzene,^7*b*,13^ and tetraphenylethene.^14^ Recently, we have developed an FML DMOS-BrTn that exhibits room-temperature phosphorescence (RTP) in its solvent-free liquid state,^15,16^ by introducing two dimethyl(octyl)silyl (DMOS) groups into an RTP-active thienyl diketone core.^17,18^ Importantly, in these alkylated FMLs, a large entropic gain from the conformational degree of freedom in the alkyl chains benefits the liquefaction. Therefore, if the degree of freedom in the alkyl chains is modulated by an external stimulus, phase behavior would be controlled, providing various FML-based stimulus-responsive systems.

We envisioned that complexation of the FMLs' alkyl chain moieties with cyclic host molecules would reduce the conformational degree of freedom and induce solidification, thereby leading to a drastic change in the functions (Fig. 1). Moreover, stimuli that disrupt the host–guest interaction would trigger decomplexation and phase separation, realizing reversible stimulus-responsive functional systems. The best candidate for this purpose would be pillar[*n*]arenes (P*n*As), which are composed of dialkoxybenzene pillars linked at the *para*-position with methylene bridges.^19^ Due to the electron-rich nature of the dialkoxybenzene units, the cavity favors the inclusion of alkanes with multiple C–H⋯π interactions. Notably, P*n*As reversibly form host–guest complexes with linear or branched alkanes depending on the ring size *n*, even in the crystalline solid states.^20^ Therefore, the host–guest chemistry of P*n*As with alkylated FMLs would be a promising platform for stimulus-responsive multicomponent systems.

Herein, we demonstrate a proof-of-concept study of the FML–P*n*A stimulus-responsive miscibility-switching system. By mixing with solid perethoxy pillar[5]arene P5A,^20*a*^ the liquid DMOS-BrTn spontaneously solidified due to the inclusion of the octyl chains in P5A. Surprisingly, the color changed from yellow/white to red, and the RTP of liquid DMOS-BrTn turned off. Crystal structure analysis revealed the successful formation of the DMOS-BrTn–P5A pseudo[3]rotaxane (1 : 2) complex, with evident inter-complex charge-transfer (CT) interactions between electron-donating P5A and the electron-accepting diketone core of DMOS-BrTn. Moreover, exposure to linear alkane vapor induced decomplexation and solid–liquid phase separation, resulting in the bleaching of the red color and the RTP turn-on. The real-time microscopic observation of the vapor responses further visualized the vapor-induced extrusion of liquid DMOS-BrTn, as well as the abrupt movements and morphological changes of the crystals. Our work paved the way for an intuitive design of multicomponent stimulus-responsive systems based on complexation–phase separation behavior of FML–cyclic host molecules.

## Results and discussion

### Synthesis and structure of the FML–P*n*A complex

**Fig. 2:**
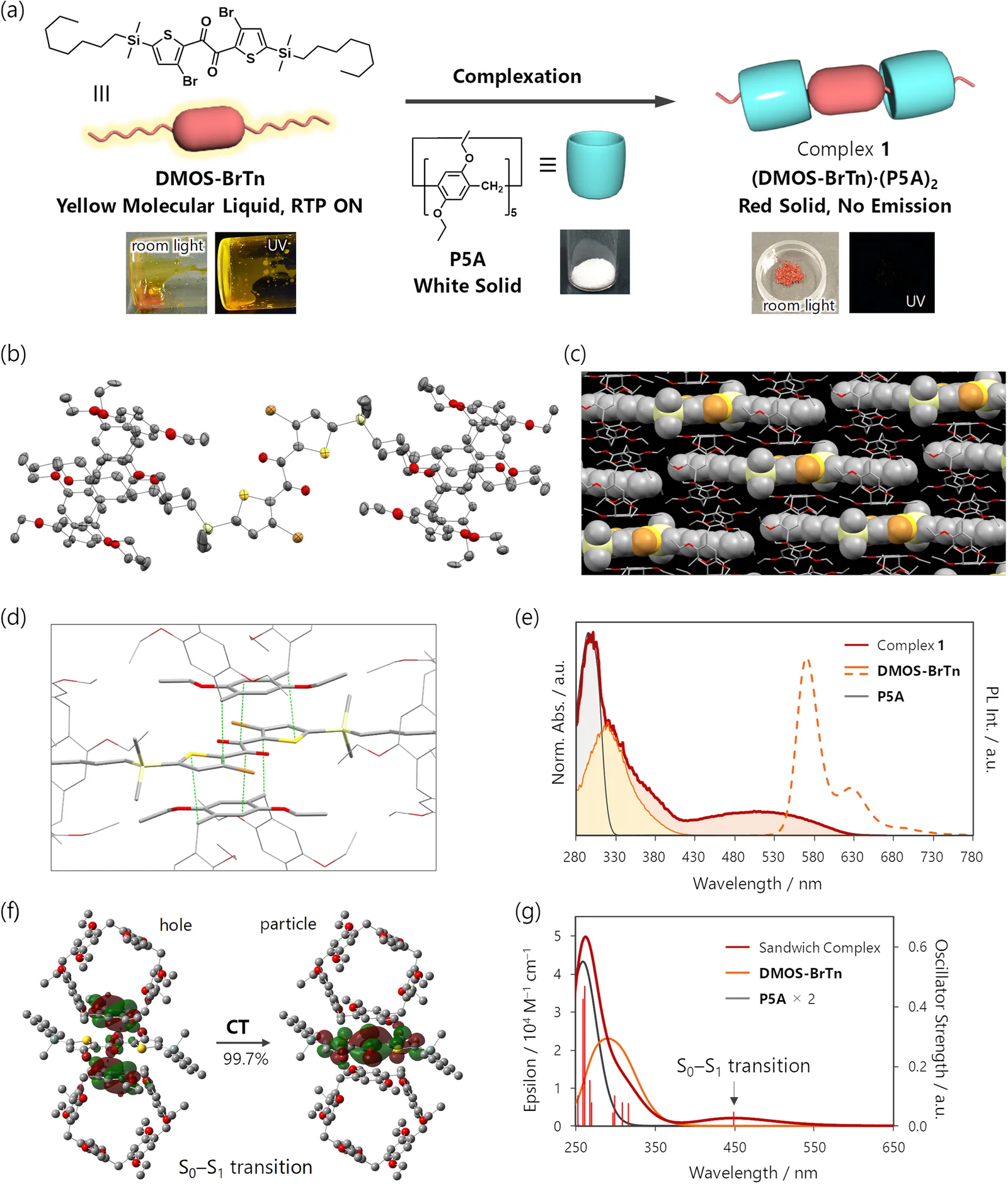
(a) Schematic showing the complexation of DMOS-BrTn with P5A and the corresponding photographs under ambient light and UV light (365 nm). (b) ORTEP drawing of the MicroED structure of complex 1. Hydrogen atoms were omitted for clarity and thermal ellipsoids were drawn at 50% probability level. (c) and (d) Crystal packing of 1. Green dotted lines represent short contacts below the sum of van der Waals radii. (e) Absorption spectra of solvent-free liquid DMOS-BrTn (orange), solid P5A (grey), and solid 1 (red), and the steady-state PL spectrum of solvent-free liquid DMOS-BrTn (orange dashed line). (f) S_0_–S_1_ natural transition orbitals for the 1 : 2 sandwich complex. (g) Simulated absorption spectra of the complex (red), DMOS-BrTn (orange), and P5A (grey), calculated at the CAM-B3LYP-D3/6-31G(d) level of theory using the crystal structure. Red bars in panel (g) represent the oscillator strength of the complex.

By mixing the white solid P5A with the yellow liquid DMOS-BrTn (three equivalents) without additional solvents, a red solid was spontaneously formed (Fig. S1). We used isopropyl alcohol to isolate the solid, because it can wash out the remaining DMOS-BrTn but does not dissolve the solid (Table S1). Namely, after adding isopropyl alcohol to the mixture, the precipitates were collected by centrifugation, washed with isopropyl alcohol, and dried under reduced pressure to provide a red crystalline solid 1 (Fig. 2a). The drastic color change suggested a successful complexation with intermolecular electronic interactions between DMOS-BrTn and P5A. Moreover, in contrast to the yellow RTP of DMOS-BrTn,^15^ no emission was observed for the red solid 1 (*vide infra*). The ^1^H NMR spectrum of 1 dissolved in CDCl_3_ indicated that, while DMOS-BrTn and P5A do not form a complex in the dilute CDCl_3_ solution,^21^1 consisted of DMOS-BrTn and P5A in a 1 : 2 molar ratio (Fig. S2). Thus, the chemical composition of 1 was assigned as (DMOS-BrTn)·(P5A)_2_. Considering that DMOS-BrTn has two octyl chains and P5A hosts one alkyl chain inside the cavity, the 1 : 2 molar ratio in 1 can be attributed to the formation of the inclusion complex in the solid state.

Although we were unable to obtain a single crystal suitable for X-ray structure analysis, the crystal structure of 1 was successfully revealed by microcrystal electron diffraction (MicroED) analysis, which is a suitable technique for sub-micrometer-sized crystals (Fig. 2b–d, S5 and Tables S2, S3).^22^ As expected, the two octyl chains of DMOS-BrTn are threaded into the P5A cavity and extended in the all-*anti* conformation, forming a dumbbell-shaped pseudo[3]rotaxane complex (Fig. 2b). More interestingly, this complex is stacked in an interdigitated manner (Fig. 2c); the central diketone moiety exhibits a planar conformation, and this electron-deficient π-plane is stacked with the electron-rich pillar of P5A in another complex (Fig. 2d). Consequently, the crystal is packed tightly without any solvent molecules. In addition, the pillar benzene unit is positioned above and below the carbonyl moiety with the closest carbon–carbon distance of ∼3.4 Å,^23^ suggestive of CT interactions.

To further validate that the whole solid consists of complex 1, powder X-ray diffractometry (PXRD) was conducted. Complex 1 showed a pattern similar to the calculated one from the MicroED crystal structure, while it was distinct from that of P5A (Fig. S4).^20*a*^ Notably, complex 1 was also obtained with a slight excess of DMOS-BrTn (DMOS-BrTn : P5A = 1.1 : 2) using CHCl_3_ as the solvent; the PXRD pattern and molar ratio of the thus obtained complex were consistent with those obtained by the solvent-free method (Fig. S3 and S4). These results strongly support the chemical identity of 1 as the DMOS-BrTn–P5A inclusion complex.

### Photophysical property changes upon complexation

Next, we investigated the optical-property changes upon complexation (Fig. 2e). Kubelka–Munk-converted diffuse reflectance spectra of crystalline P5A (guest-free) and liquid DMOS-BrTn had a maximum at 296 nm and around 320 nm, respectively. In contrast, the spectrum of complex 1 exhibited a new broad band spanning from 420 to 650 nm with a maximum at 510 nm. The spectral feature in the shorter wavelength region can be interpreted as the sum of those of P5A and DMOS-BrTn. The emergence of the new band is responsible for the drastic color change from the yellow liquid and white solid to the red solid (Fig. 2a). This emerged band is ascribed to the CT absorption between the donor diethoxybenzene units of P5A and the acceptor thienyl diketone core of DMOS-BrTn.

To unveil the nature of the new absorption band, we extracted the geometry of DMOS-BrTn along with that of two stacked P5A molecules from the MicroED crystal structure and performed single-point time-dependent density-functional theory (TDDFT) calculations at the CAM-B3LYP-D3/6-31G(d) level. The simulated absorption spectrum excellently reproduced the experimental one (Fig. 2g). The longer wavelength absorption was revealed to originate from the S_0_–S_1_ transition, which demonstrates clear CT from P5A to DMOS-BrTn, according to natural transition orbital analysis (Fig. 2f). The occurrence of the CT interaction can be predicted by simply calculating the energy-level alignment of FML and P*n*A (Fig. S7). The HOMO level of permethoxy P5A was higher than that of DMOS-BrTn, while the LUMO level of DMOS-BrTn was lower than that of permethoxy P5A. The gap between the HOMO of permethoxy P5A and the LUMO of DMOS-BrTn was 2.01 eV, which was well below the absorption energy (510 nm, 2.43 eV).

The complexation of DMOS-BrTn with P5A not only brought a drastic color change but also turned off the RTP (Fig. 2a). The RTP of liquid DMOS-BrTn was observed at 571 nm with a decent quantum yield of 5.6% in air (Fig. 2e),^15^ while that of complex 1 was invisible and too weak for quantum yield measurement. This is intriguing because encapsulating phosphors with a cyclic host often result in RTP turn-on or enhancement,^24,25^ with rare exceptions.^26^ Moreover, in the present system, complexation-induced solidification of the liquid phosphor, which would rigidify the molecular environment and suppress nonradiative decay, is expected to increase the RTP efficiency.^27–29^

The RTP turn-off is likely because the newly formed CT state is a dark state. To test this hypothesis, we conducted a quenching experiment on the RTP intensity and lifetime of DMOS-BrTn in cyclohexane using 2,5-dimethoxytoluene P1 as a structurally simpler model quencher of P5A (Fig. S8a). The obtained Stern–Volmer plot revealed that P1 quenches the lowest triplet (T_1_) state of DMOS-BrTn with a bimolecular rate constant *k*_q_ = 3.7 × 10^8^ M^−1^ s^−1^ (Fig. S8b), probably *via* the formation of a dark exciplex.^30^ According to TDDFT calculations, the T_1_ energy level of P1 (3.67 eV) is much higher than that of DMOS-BrTn (2.11 eV), excluding Dexter energy transfer from DMOS-BrTn. These results suggest that the CT state of DMOS-BrTn–P5A complex 1 is also a dark state, causing the RTP turn-off. We note that the spectral region of RTP from the liquid DMOS-BrTn mostly overlaps that of the absorption of the crystalline DMOS-BrTn–P5A complex 1. This fact may imply a possibility of a self-absorption-caused RTP turn-off. However, microcrystals, which are supposed to have minimal self-absorption, also show no RTP according to observations under a photoluminescence microscope (*vide infra*). Therefore, we conclude that the RTP turn-off is due to the formation of the dark CT state rather than the self-absorption mechanism.

### Vapor-responses of DMOS-BrTn–P5A complex 1

**Fig. 3:**
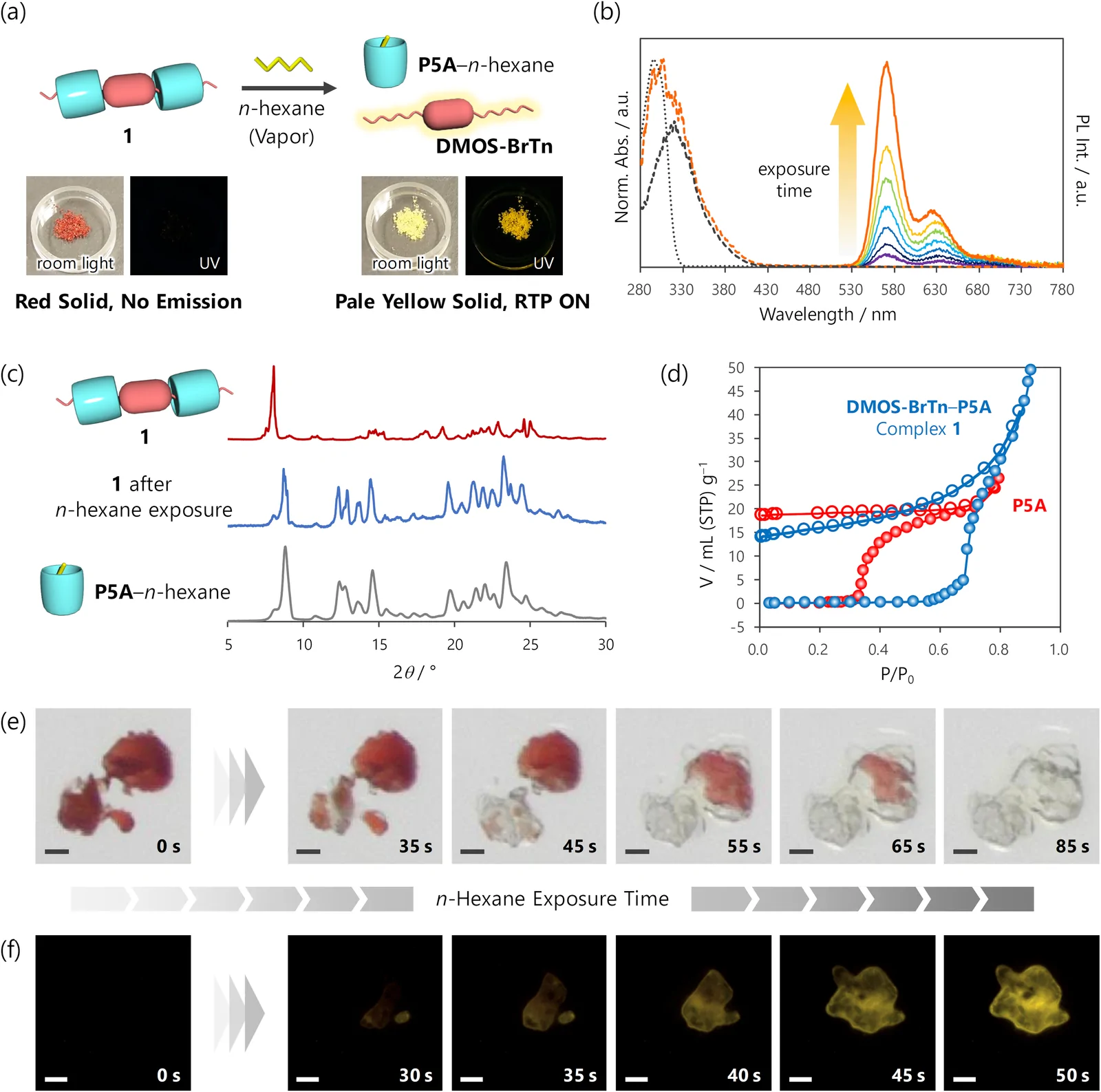
(a) The *n*-hexane vapor response of DMOS-BrTn–P5A complex 1 and the corresponding photographs under ambient light and UV light (365 nm). (b) Absorption (dashed/dotted lines) and PL spectral change of 1 upon exposure of *n*-hexane vapor (solid lines). Orange dashed line: 1 after exposure; black dashed line: DMOS-BrTn; black dotted line: P5A. (c) PXRD patterns of 1 before (red line) and after exposure to *n*-hexane vapor (blue line) and the P5A–*n*-hexane complex (grey line). (d) Sorption isotherms of P5A (red line) and 1 (blue line) towards *n*-hexane vapor at 25 °C. Solid symbols: adsorption; open symbols: desorption. (e) and (f) Images captured from the real-time observation of 1 upon exposure to *n*-hexane vapor under (e) the optical and (f) luminescence microscope excited at 405 nm. Scale bar = 20 µm (e) and 5 µm (f).

The crystalline DMOS-BrTn–P5A complex 1 exhibited drastic property changes in response to *n*-hexane vapor (Fig. 3). Namely, when the solid was placed in a closed glass container and exposed to *n*-hexane vapor, which is a good guest for crystalline P5A,^20*a*,*d*^ the color of the solid reverted from red to yellowish white, and the yellow emission turned on (Fig. 3a, S9 and SI Movie S1). The Kubelka–Munk-converted diffuse reflectance spectra demonstrated the disappearance of the broad absorption band at 420–650 nm, confirming the loss of the CT interactions (Fig. 3b). In addition, the time course of the photoluminescence spectra during vapor exposure clearly showed the emergence of emission at 571 nm. The spectral shape was identical to that of RTP from liquid DMOS-BrTn,^15^ and the emission lifetime was 2.8 µs in air (Fig. S11). Therefore, the observed phenomenon was a vapor-induced turn-on RTP.^18*a*,31^

The turning on of RTP indicates that the formation of the exciplex, as well as the ground-state CT complex, was inhibited after exposure to *n*-hexane vapor. Moreover, the excitation spectrum of the complex after the vapor exposure exhibited a deep dip in the P5A absorption region. The dip is indicative of the inefficient energy transfer from P5A to DMOS-BrTn (Fig. S12). These optical properties suggest that, after the exposure to *n*-hexane vapor, DMOS-BrTn is no longer molecularly dispersed in P5A but rather segregated to form a separate phase, *i.e.*, the color change and RTP turn-on were based on vapor-induced solid–liquid phase separation.

To gain insight into the vapor-induced structural change, we examined PXRD of 1 after exposing it to *n*-hexane vapor (Fig. 3c). The obtained profile was distinct from the original one and was identical to that of the crystalline P5A–*n*-hexane inclusion complex.^20*a*^ Moreover, DMOS-BrTn was not discernible, which is reasonable as liquid DMOS-BrTn only showed a weak and broad halo.^15^ These results strongly suggest the concomitant crystal transformation and solid–liquid phase separation; *n*-hexane replaced the octyl chains of DMOS-BrTn to fill in the cavity of P5A, thereby forming a P5A–*n*-hexane inclusion complex crystal and extruding liquid DMOS-BrTn out of the crystal.

Furthermore, we measured the *n*-hexane sorption isotherm of 1 (Fig. 3d, blue symbols). In the adsorption process, 1 did not take up the vapor at low pressure. Around a partial pressure of 0.7, a sudden uptake occurred. This response is referred to as gate-opening behavior, indicating a crystal structure change.^20*a*,*c*,32^ The gate-opening pressure was larger than that observed in the *n*-hexane sorption isotherm of guest-free P5A crystals (around 0.3; Fig. 3d, red symbols). These results are in good agreement with the crystal structure, in which the octyl chains reside in the cavity of P5A, acting as a competing guest for *n*-hexane.

To corroborate the crystal transformation and the solid–liquid phase separation, we performed real-time observation of the crystalline DMOS-BrTn–P5A complex 1 during *n*-hexane vapor exposure under an optical microscope (Fig. 3e and SI Movies S2–S5). The gradual bleaching of the red color clearly visualized the progress of the vapor response. Meanwhile, the liquid seeped out from the crystal, which corresponded to the solid–liquid phase separation. Real-time observation was also conducted under a photoluminescence microscope (Fig. 3f and SI Movie S6). The seeped-out liquid beneath the crystal exhibited yellow emission, further confirming the vapor-induced solid–liquid phase separation.

Interestingly, crystals moved and reshaped abruptly during the microscopic observation. More drastic movements, such as jumping, cracking, or separation of debris, of organic crystals upon applying external stimulus have been reported and are called thermo-,^33^ photo-,^34^ or mechanosalient effects^35^ (heat, light, or mechanical stimulus, respectively).^36^ Although the present phenomenon is relatively modest, we may refer to these abrupt, non-gradual movements as a vaposalient effect.^37^

### Selectivity and reversibility of the vapor response

**Fig. 4:**
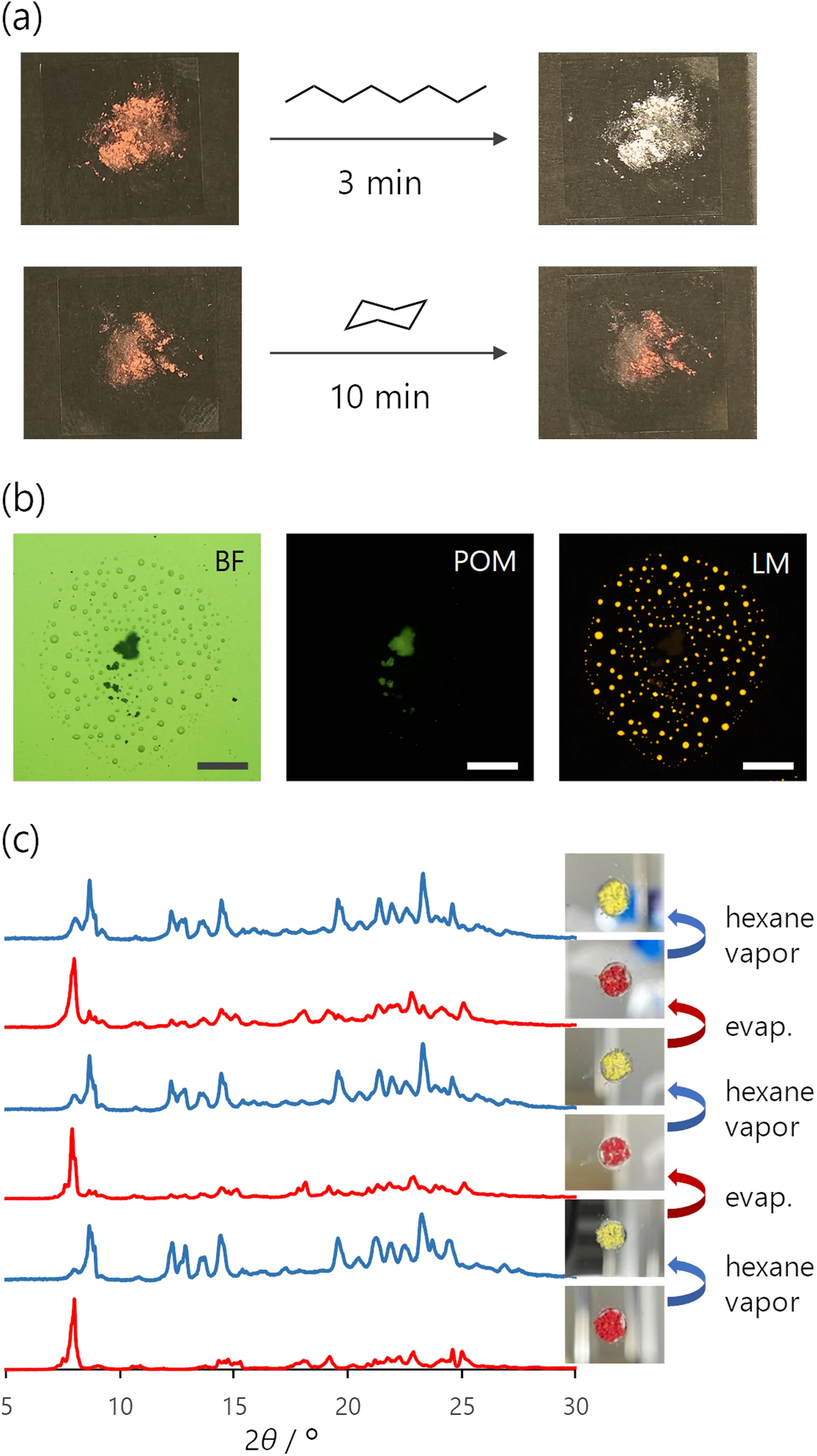
(a) Photographic images of 1 before and after exposing to the vapor of octane (top) and cyclohexane (bottom) for the indicated time. (b) Microscopic images of 1 after exposing hexane vapor on a flat surface. Images were taken under: BF, bright-field; POM, polarizing optical microscope under a cross Nichol prism; LM, luminescence microscope under 365 nm excitation. Scale bar = 0.1 mm. (c) PXRD profiles and the corresponding photographic images of 1 after exposing to hexane vapor (blue lines) and after evaporating under reduced pressure at 60 °C (red lines).

The response of 1 was specific to the molecular shape of the vapor. Octane, a linear alkane, induced the same response in color and RTP properties, while cyclohexane, a cyclic alkane, did not lead to any change after a prolonged exposure (Fig. 4a and S13). Such selectivity was reminiscent of guest-free P5A crystals, which take up *n*-alkanes but do not take up branched and cyclic alkanes that are too large to fit into the cavity.^20*a*^ Therefore, the vapor-selective response originated from the molecular recognition ability of P5A. Importantly, this selectivity clearly indicates that the vapor response of 1 was not due to the extraction of DMOS-BrTn by the solvent vapor. To the best of our knowledge, this is the first example of the vapor-shape selective turn-on RTP phenomenon.

The reversibility of the vapor response can be altered based on the solid–liquid phase separation (Fig. 4b and c). When a small amount of 1 was placed on a flat surface and exposed to hexane vapor, the liquid was extruded and was distant from the solid (Fig. 4b). The polarized optical microscope image under a cross Nichol prism indicated that the central solid was crystalline, while the surrounding liquid was isotropic. The microscopic image under UV irradiation visualized that the crystalline solid was nonemissive, while the liquid droplets exhibited yellow emission. Thus, the extruded liquid and the crystalline solid are assignable to DMOS-BrTn and the P5A–hexane complex, respectively. The transportation of the liquid was likely aided by the absorption–evaporation cycle of hexane, which temporarily increased the fluidity of the liquid during vapor exposure.

In contrast, when a certain amount of crystalline powder was mounded and exposed to vapor, the solid part exhibited RTP, indicating that liquid DMOS-BrTn remained absorbed in the crystalline powder of P5A–hexane complex (Fig. 3a). To evaluate the reversibility, the crystalline DMOS-BrTn–P5A complex 1 was placed in a well for PXRD (*ϕ* = 5 mm; depth: 0.1 mm) and exposed to *n*-hexane vapor. The red color turned yellow, and the PXRD pattern changed to that of the P5A–hexane complex (Fig. 4c). The color and the PXRD pattern reverted to their initial state after drying under reduced pressure for 12 h at 60 °C. This cycle was repeated 3 times without the loss of reversibility. This reversible response is probably due to the extruded DMOS-BrTn being retained between the crystalline domains of the P5A–hexane complex.

## Conclusions

We have established a stimulus-responsive miscibility switching of an FML–cyclic host bicomponent system. Namely, linear alkane vapor mediates the reversible interconversion between a single-phase solid state and a solid–liquid phase-separated state. This results in drastic chromism and RTP responses. The phase control largely alters the distance between the donor P5A and the acceptor DMOS-BrTn, resulting in the distinct ON–OFF switching of the donor–acceptor interaction, thereby achieving significant optical-property responses. The key to this approach is exploiting the encapsulation/extrusion of alkyl chain by P*n*As; this concept would in principle be applicable to a wide range of alkylated FMLs, considering that the entropic gain from the alkyl chain plays a crucial role in liquifying them. Moreover, our work illustrates that molecular-scale host–guest chemistry induces macroscopic phase separation, which would stimulate further advancement in supramolecular chemistry and materials science.

## Author contributions

Y. T. and T. O. designed the project. K. W. and Y. O. synthesized and characterized the compounds. Y. T. and Y. O. evaluated the photophysical properties. T. N. and A. K. performed MicroED. T. T. performed real-time observations of the vapor response under a microscope. Y. T. performed theoretical calculations. Y. T. and K. W. wrote the manuscript with contributions from all authors. Y. T. and K. W. contributed equally.

## Conflicts of interest

There are no conflicts to declare.

## Supplementary Material

SC-017-D6SC03713E-s001

SC-017-D6SC03713E-s002

SC-017-D6SC03713E-s003

SC-017-D6SC03713E-s004

SC-017-D6SC03713E-s005

SC-017-D6SC03713E-s006

SC-017-D6SC03713E-s007

SC-017-D6SC03713E-s008

SC-017-D6SC03713E-s009

## Data Availability

The data supporting this article have been included as part of the supplementary information (SI). Supplementary information is available. See DOI: https://doi.org/10.1039/d6sc03713e. Raw MicroED datasets (diffraction images) have been deposited to XRDa (https://xrda.pdbj.org/; accession code XRD-387). Refined coordinates of 1 have been deposited to CCDC (2504535)^22^ and COD (3000627). Detailed data collection protocols and SerialEM macros are available at our GitHub repository (https://github.com/GKLabIPR/MicroED).
