# Supplementary material for: Vapor-induced miscibility switching and optical response in a functional molecular liquid–pillar[5]arene system
Source: Chem Sci. 2026 Jul 21. Online ahead of print. doi: 10.1039/d6sc03713e (PMC13386138; doi:10.1039/d6sc03713e)
Supplement: SC-OLF-D6SC03713E-s001 [file SC-OLF-D6SC03713E-s001.pdf]

Supplementary Information (SI) for Chemical Science.
This journal is © The Royal Society of Chemistry 2026

C1P5A @S_0

E(RB3LYP)	-2497.740445	a.u.	
C	0	4.979828	0
H	-0.00615	5.624862	0.880069
H	0.006153	5.624862	-0.88007
C	4.736098	1.538851	0
H	5.347661	1.74403	0.880069
H	5.351464	1.732326	-0.88007
C	2.927069	-4.02877	0
H	3.301233	-4.55423	-0.88007
H	3.311189	-4.54699	0.880069
C	-2.92707	-4.02877	0
H	-3.31119	-4.54699	-0.88007
H	-3.30123	-4.55423	0.880069
C	-4.7361	1.538851	0
H	-5.35146	1.732326	0.880069
H	-5.34766	1.74403	-0.88007
C	1.252865	4.125926	0.006082
C	1.828296	3.705945	-1.19302
H	1.350202	4.020384	-2.11074
C	2.949608	2.876749	-1.20949
C	3.536833	2.466526	-0.00608
C	2.959589	2.884013	1.193016
C	1.824471	3.694208	1.209489
C	4.311145	0.083436	0.006082
C	4.089538	-0.59361	-1.19302
H	4.240848	-0.04175	-2.11074
C	3.647429	-1.91628	-1.20949
C	3.438747	-2.60153	-0.00608
C	3.657422	-1.92353	1.193016
C	4.077193	-0.5936	1.209489
C	1.41157	-4.07436	0.006082
C	0.699178	-4.07282	-1.19302
H	1.270786	-4.04619	-2.11074
C	-0.69537	-4.06108	-1.20949
C	-1.41157	-4.07436	-0.00608

C	-0.69918	-4.07282	1.193016
C	0.695373	-4.06108	1.209489
C	-3.43875	-2.60153	0.006082
C	-3.65742	-1.92353	-1.19302
H	-3.45546	-2.45893	-2.11074
C	-4.07719	-0.5936	-1.20949
C	-4.31115	0.083436	-0.00608
C	-4.08954	-0.59361	1.193016
C	-3.64743	-1.91628	1.209489
C	-3.53683	2.466526	0.006082
C	-2.95959	2.884013	-1.19302
H	-3.40638	2.526486	-2.11074
C	-1.82447	3.694208	-1.20949
C	-1.25287	4.125926	-0.00608
C	-1.8283	3.705945	1.193016
C	-2.94961	2.876749	1.209489
O	3.539183	2.412678	-2.36528
C	2.959471	2.776978	-3.60364
H	2.979562	3.862178	-3.75818
H	1.924372	2.424078	-3.6849
O	3.388261	-2.6204	-2.36528
C	3.55559	-1.95649	-3.60364
H	4.593885	-1.64025	-3.75818
H	2.900099	-1.08111	-3.6849
O	-1.44512	-4.03218	-2.36528
C	-0.762	-3.98616	-3.60364
H	-0.14039	-4.87591	-3.75818
H	-0.13201	-3.09224	-3.6849
O	-4.2814	0.128382	-2.36528
O	-1.20093	4.111521	-2.36528
C	-1.72654	3.672758	-3.60364
H	-2.75241	4.027211	-3.75818
H	-1.71077	2.579268	-3.6849
C	-4.02653	-0.50709	-3.60364
H	-4.68065	-1.37323	-3.75818
H	-2.98169	-0.83	-3.6849

H	-1.27079	-4.04619	2.110738
H	-4.24085	-0.04175	2.110738
H	3.455459	-2.45893	2.110738
H	-1.08273	4.101054	-4.37142
H	-4.23492	0.237555	-4.37142
H	3.565751	2.297036	-4.37142
H	3.286488	-2.68141	-4.37142
H	-1.53459	-3.95424	-4.37142
O	4.281396	0.128382	2.365283
O	1.200926	4.111521	2.365283
O	-3.53918	2.412678	2.365283
O	-3.38826	-2.6204	2.365283
O	1.445122	-4.03218	2.365283
C	-3.55559	-1.95649	3.603636
H	-3.28649	-2.68141	4.371423
H	-2.9001	-1.08111	3.6849
H	-4.59389	-1.64025	3.75818
C	0.761996	-3.98616	3.603636
H	1.534589	-3.95424	4.371423
H	0.132012	-3.09224	3.6849
H	0.140385	-4.87591	3.75818
C	4.026529	-0.50709	3.603636
H	4.234917	0.237555	4.371423
H	2.981687	-0.83	3.6849
H	4.680648	-1.37323	3.75818
C	1.726536	3.672758	3.603636
H	1.082733	4.101054	4.371423
H	1.710772	2.579268	3.6849
H	2.752414	4.027211	3.75818
C	-2.95947	2.776978	3.603636
H	-3.56575	2.297036	4.371423
H	-1.92437	2.424078	3.6849
H	-2.97956	3.862178	3.75818
H	3.406377	2.526486	2.110738
H	-1.3502	4.020384	2.110738

DMOS-BrTn @S_0

E(RB3LYP)	-7846.768141	a.u.	
C	3.314667	2.452397	1.260679
C	3.966895	1.644507	0.355366
S	2.870494	0.507373	-0.35134
C	1.503097	1.113493	0.573488
C	1.93862	2.15709	1.383518
C	0.136275	0.587706	0.487345
O	-0.7837	1.037023	1.147493
C	-0.13628	-0.58771	-0.48735
O	0.783696	-1.03702	-1.14749
C	-1.5031	-1.11349	-0.57349
C	-1.93862	-2.15709	-1.38352
C	-3.31467	-2.4524	-1.26068
C	-3.9669	-1.64451	-0.35537
S	-2.87049	-0.50737	0.351341
Br	0.877524	3.171041	2.587321
Br	-0.87752	-3.17104	-2.58732
H	3.79341	3.239073	1.829208
H	-3.79341	-3.23907	-1.82921
Si	5.798872	1.689067	-0.1085
Si	-5.79887	-1.68907	0.108496
C	-5.94554	-2.04129	1.952399
H	-6.99336	-2.09558	2.262859
H	-5.47161	-2.99096	2.215476
H	-5.46538	-1.25877	2.547368
C	-6.60574	-3.0576	-0.90095
H	-6.15078	-4.03172	-0.70006
H	-7.66919	-3.13969	-0.65815
H	-6.52927	-2.86759	-1.97542
C	-6.54786	-0.00359	-0.29951
H	-5.97473	0.766644	0.233095
H	-6.38532	0.197878	-1.36598
C	6.605744	3.057599	0.900954
H	7.669186	3.139689	0.658146
H	6.150776	4.031721	0.700061

H	6.529272	2.867589	1.975421
C	5.945543	2.041287	-1.9524
H	5.471608	2.990958	-2.21548
H	6.993362	2.095582	-2.26286
H	5.465381	1.258768	-2.54737
C	6.547864	0.003585	0.299506
H	6.385323	-0.19788	1.365982
H	5.974729	-0.76664	-0.2331
C	-8.04223	0.137138	0.037446
C	-8.61153	1.518036	-0.30573
H	-8.20352	-0.05828	1.105135
H	-8.61949	-0.62773	-0.49788
C	-10.0986	1.663987	0.027362
H	-8.03899	2.286093	0.230083
H	-8.45429	1.71692	-1.37375
C	-10.6671	3.043957	-0.31501
H	-10.6687	0.893596	-0.50867
H	-10.2533	1.462759	1.095683
H	-10.0972	3.814226	0.22104
H	-10.5121	3.245518	-1.38312
C	8.042231	-0.13714	-0.03745
C	8.611526	-1.51804	0.305725
H	8.619491	0.627732	0.497881
H	8.20352	0.058282	-1.10514
C	10.09861	-1.66399	-0.02736
H	8.454286	-1.71692	1.373747
H	8.03899	-2.28609	-0.23008
C	10.66705	-3.04396	0.31501
H	10.25331	-1.46276	-1.09568
H	10.6687	-0.8936	0.508667
H	10.51212	-3.24552	1.383115
H	10.09723	-3.81423	-0.22104
C	-12.1543	3.19064	0.017536
C	-12.7244	4.570449	-0.32412
H	-12.3098	2.989111	1.085805
H	-12.7247	2.420393	-0.51851

C	-14.2109	4.705275	0.01274
H	-12.1549	5.3398	0.212028
H	-12.5698	4.771393	-1.3916
H	-14.5939	5.698019	-0.24
H	-14.8095	3.970606	-0.53541
H	-14.3914	4.543175	1.080256
C	12.15425	-3.19064	-0.01754
C	12.72437	-4.57045	0.324123
H	12.72472	-2.42039	0.51851
H	12.30979	-2.98911	-1.08581
C	14.21093	-4.70528	-0.01274
H	12.56982	-4.77139	1.391599
H	12.15485	-5.3398	-0.21203
H	14.59385	-5.69802	0.24
H	14.39138	-4.54318	-1.08026
H	14.8095	-3.97061	0.535411
H	-4.59389	-1.64025	3.75818
C	0.761996	-3.98616	3.603636
H	1.534589	-3.95424	4.371423
H	0.132012	-3.09224	3.6849
H	0.140385	-4.87591	3.75818
C	4.026529	-0.50709	3.603636
H	4.234917	0.237555	4.371423
H	2.981687	-0.83	3.6849
H	4.680648	-1.37323	3.75818
C	1.726536	3.672758	3.603636
H	1.082733	4.101054	4.371423
H	1.710772	2.579268	3.6849
H	2.752414	4.027211	3.75818
C	-2.95947	2.776978	3.603636
H	-3.56575	2.297036	4.371423
H	-1.92437	2.424078	3.6849
H	-2.97956	3.862178	3.75818
H	3.406377	2.526486	2.110738
H	-1.3502	4.020384	2.110738

DMOS-BrTn @T_1

E(UB3LYP)	-7846.695483	a.u.	
C	3.2871	2.442519	1.255741
C	3.947685	1.638021	0.354579
S	2.852913	0.488312	-0.36122
C	1.48724	1.096661	0.561369
C	1.912534	2.142689	1.373458
C	0.143387	0.574026	0.471613
O	-0.79922	1.034648	1.145646
C	-0.14339	-0.57403	-0.47161
O	0.799218	-1.03465	-1.14565
C	-1.48724	-1.09666	-0.56137
C	-1.91253	-2.14269	-1.37346
C	-3.2871	-2.44252	-1.25574
C	-3.94769	-1.63802	-0.35458
S	-2.85291	-0.48831	0.361223
Br	0.807012	3.129848	2.561201
Br	-0.80701	-3.12985	-2.5612
H	3.763811	3.230925	1.824315
H	-3.76381	-3.23093	-1.82432
Si	5.775948	1.679515	-0.10891
Si	-5.77595	-1.67952	0.108905
C	-5.93154	-2.03221	1.952345
H	-6.98066	-2.08575	2.258689
H	-5.45911	-2.98223	2.216803
H	-5.45344	-1.25051	2.550201
C	-6.57909	-3.04909	-0.90315
H	-6.12388	-4.0228	-0.70101
H	-7.64295	-3.13154	-0.66223
H	-6.50046	-2.85865	-1.97734
C	-6.53031	0.004096	-0.29847
H	-5.9584	0.776406	0.232614
H	-6.36925	0.205044	-1.36526
C	6.579087	3.049089	0.903149
H	7.642953	3.131536	0.662227
H	6.123881	4.022802	0.701012

H	6.500455	2.858646	1.977337
C	5.931544	2.032205	-1.95235
H	5.459109	2.982232	-2.2168
H	6.980655	2.085752	-2.25869
H	5.453442	1.25051	-2.5502
C	6.530314	-0.0041	0.298471
H	6.36925	-0.20504	1.365258
H	5.958395	-0.77641	-0.23261
C	-8.02447	0.14255	0.040181
C	-8.59721	1.521599	-0.3047
H	-8.18407	-0.05139	1.108389
H	-8.60089	-0.62445	-0.49305
C	-10.0842	1.665175	0.029773
H	-8.02566	2.291835	0.229077
H	-8.4416	1.718991	-1.37324
C	-10.6559	3.043327	-0.31457
H	-10.6533	0.892612	-0.50418
H	-10.2373	1.465608	1.098634
H	-10.087	3.815772	0.21944
H	-10.5024	3.243204	-1.3832
C	8.02447	-0.14255	-0.04018
C	8.597212	-1.5216	0.304699
H	8.600888	0.624451	0.493049
H	8.18407	0.05139	-1.10839
C	10.08422	-1.66518	-0.02977
H	8.441597	-1.71899	1.373239
H	8.025656	-2.29184	-0.22908
C	10.65587	-3.04333	0.314567
H	10.23734	-1.46561	-1.09863
H	10.65333	-0.89261	0.504177
H	10.50243	-3.2432	1.383204
H	10.08705	-3.81577	-0.21944
C	-12.143	3.187695	0.019194
C	-12.7162	4.565712	-0.32451
H	-12.2971	2.987876	1.088
H	-12.7125	2.415297	-0.51481

C	-14.2027	4.698271	0.013574
H	-12.1477	5.337215	0.209593
H	-12.5631	4.764925	-1.39252
H	-14.5879	5.689767	-0.2407
H	-14.8004	3.961348	-0.53253
H	-14.3818	4.537886	1.081591
C	12.14302	-3.1877	-0.01919
C	12.71622	-4.56571	0.324512
H	12.71249	-2.4153	0.514813
H	12.2971	-2.98788	-1.088
C	14.20271	-4.69827	-0.01357
H	12.56314	-4.76493	1.392524
H	12.14769	-5.33722	-0.20959
H	14.58786	-5.68977	0.240696
H	14.38178	-4.53789	-1.08159
H	14.80037	-3.96135	0.532531
H	-4.59389	-1.64025	3.75818
C	0.761996	-3.98616	3.603636
H	1.534589	-3.95424	4.371423
H	0.132012	-3.09224	3.6849
H	0.140385	-4.87591	3.75818
C	4.026529	-0.50709	3.603636
H	4.234917	0.237555	4.371423
H	2.981687	-0.83	3.6849
H	4.680648	-1.37323	3.75818
C	1.726536	3.672758	3.603636
H	1.082733	4.101054	4.371423
H	1.710772	2.579268	3.6849
H	2.752414	4.027211	3.75818
C	-2.95947	2.776978	3.603636
H	-3.56575	2.297036	4.371423
H	-1.92437	2.424078	3.6849
H	-2.97956	3.862178	3.75818
H	3.406377	2.526486	2.110738
H	-1.3502	4.020384	2.110738
