# Supplementary material for: Vapor-induced miscibility switching and optical response in a functional molecular liquid–pillar[5]arene system
Source: Chem Sci. 2026 Jul 21. Online ahead of print. doi: 10.1039/d6sc03713e (PMC13386138; doi:10.1039/d6sc03713e)
Supplement: SC-OLF-D6SC03713E-s002 [file SC-OLF-D6SC03713E-s002.pdf]

Supplementary Information (SI) for Chemical Science.
This journal is © The Royal Society of Chemistry 2026

Electronic Supplementary Information for

Vapor-induced Miscibility Switching and Optical Response in a Functional Molecular Liquid–Pillar[5]arene System

Yosuke Tani,*†[a,b] Keisuke Wada,†[c] Yuya Oshima,[a] Takanori Nakane,[d,e] Akihiro Kawamoto,[d,e] Genji Kurisu,*[d,e] Takashi Tachikawa,*[f,g] Shunsuke Ohtani,[c] Kenichi Kato,[c] and Tomoki Ogoshi*[c,h]

[a]Department of Chemistry, Graduate School of Science, The University of Osaka, Machikaneyama 1-1, Toyonaka, Osaka 560-0043, Japan; [b]Institute of Transformative Bio-Molecules (ITbM), Nagoya University, Furo, Chikusa, Nagoya 464-8601, Japan; [c]Department of Synthetic Chemistry and Biological Chemistry, Graduate School of Engineering, Kyoto University, Katsura, Nishikyo-ku, Kyoto 615-8510, Japan; [d]Institute for Protein Research, The University of Osaka, 3–2 Yamadaoka, Suita, Osaka 565-0871, Japan; [e]JEOL YOKOGUSHI Research Alliance Laboratories, Graduate School of Frontier Biosciences, The University of Osaka, 1-3 Yamadaoka, Suita, Osaka 565-0871 Japan; [f]Center for Life Photonic Innovation, Kobe University, 1-1 Rokkodai-cho, Nada-ku, Kobe 657-8501, Japan; [g]Department of Chemistry, Graduate School of Science, Kobe University, 1-1 Rokkodai-cho, Nada-ku, Kobe 657-8501, Japan; [h]WPI Nano Life Science Institute (WPI-Nano LSI), Kanazawa University, Kakuma-machi, Kanazawa 920-1192, Japan

E-mail: tani.yosuke.y1@f.mail.nagoya-u.ac.jp

Table of Contents

1. Instrumentation and Chemicals 2
2. Preparation and Characterization of Complex 1 2
3. MicroED Data Collection and Processing 5
4. Theoretical Calculations 9
5. Photophysical Properties 10
6. References 16

1. Instrumentation and Chemicals

Centrifugation was performed on a centrifuge KOKUSAN H-36α. All the measurements were performed at room temperature (RT) in air unless otherwise noted. ^{1}H NMR spectra were recorded on a JEOL JNM-ECS400 or JNM-ECZ500R spectrometer. Chemical shift values (δ) are reported in ppm and are calibrated to tetramethylsilane (0.00 ppm). UV-vis absorption spectra were obtained by the Kubelka–Munk conversion of diffuse reflectance spectra acquired using a Shimadzu UV-3150 or a Hitachi U-4100 spectrometer with an integrating sphere. Steady-state photoluminescence (PL) and excitation spectra were acquired using a JASCO FP-8200 spectrofluorometer. PL quantum yield was determined by absolute method using a Hamamatsu photonics C9920-02 spectrometer with an integrating sphere. PL lifetime measurement was performed using a HORIBA DeltaFlex multichannel scaling system using DeltaDiode for excitation (368 nm). Powder X-ray diffraction (PXRD) measurements were performed on a Rigaku Smart Lab high-resolution diffractometer. The amount of hexane vapor adsorption was measured using a BELSORP-max-12-N-VP-K (BEL Japan Inc., Osaka, Japan).

Commercially available chemicals were used as received. 1,2-bis[3-bromo-5-(dimethyloctylsilyl)thiophen-2-yl]ethane-1,2-dione (**DMOS-BrTn**) and diethoxypillar[5]arene (**P5A**) were synthesized according to the previous reports.[1]

2. Preparation and Characterization of Complex 1

Method 1. Activated crystals of **P5A** (21 mg, 1.0 eq.) were immersed in a 5 mL vial containing **DMOS-BrTn** (50 mg, 3.0 eq.). After adding isopropyl alcohol, the precipitate was collected by centrifugation, washed with isopropyl alcohol three times, and dried *in vacuo* to give red crystalline complex **1** (25 mg, 85% yield).

Method 2. Activated crystals of **P5A** (311 mg, 1.0 eq) and an excess amount of **DMOS-BrTn** (144 mg, 0.57 eq) were dissolved in $CHCl_3$, and then $CHCl_3$ was completely evaporated and dried *in vacuo* at 60 °C. After adding isopropyl alcohol, the precipitate was collected by centrifugation, washed with isopropyl alcohol three times, and dried *in vacuo* to give red crystalline complex **1** (353 mg, 81% yield).

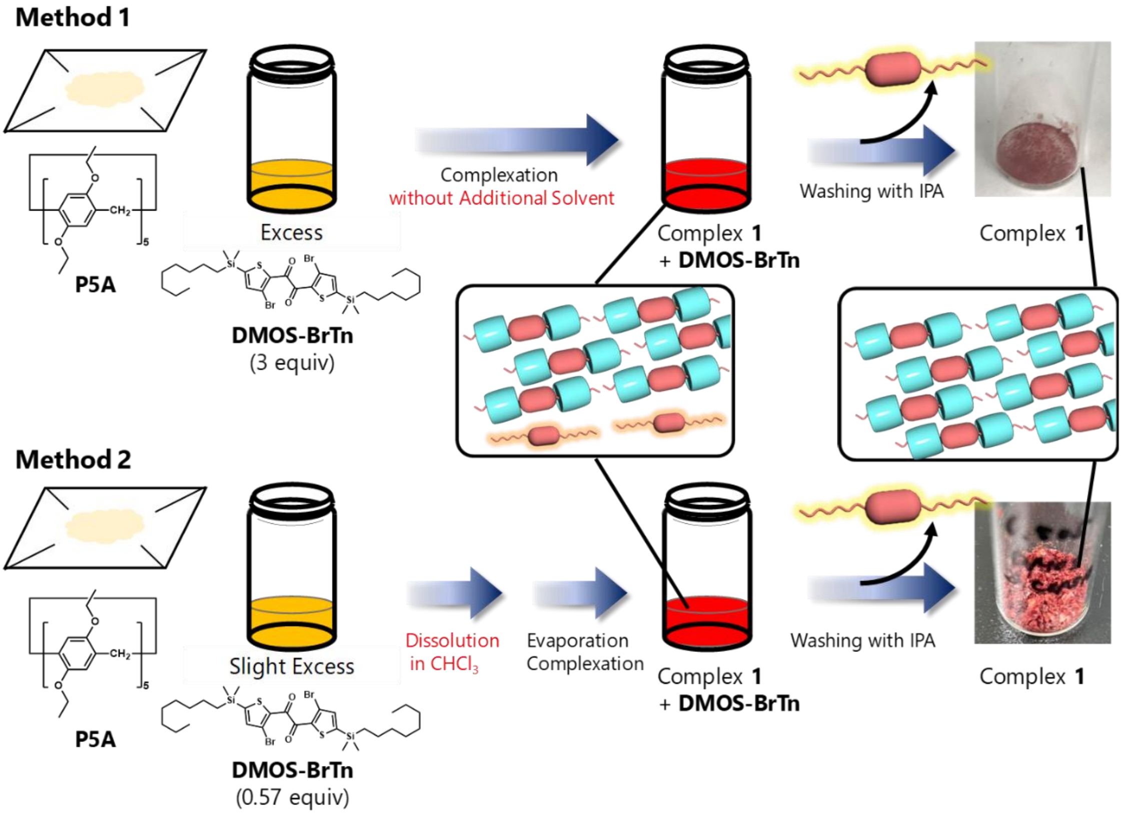

Figure S1. Schematic representation of preparing complex **1**.

Table S1. Summary of Solvent Screening Results

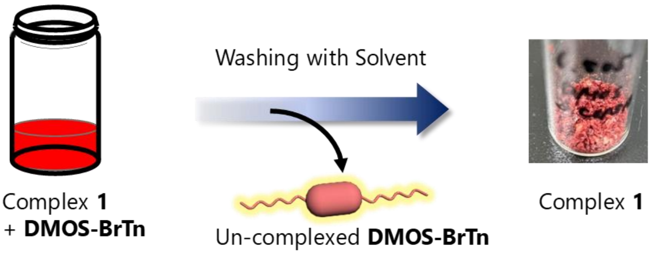

Washing Solvent	Results	
Hexane	Decomplexation	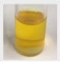
Cyclohexane	Decomplexation	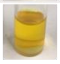
THF	Decomplexation	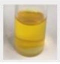
Et_2O	Decomplexation	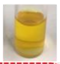
IPA	Retaining Complex	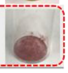

NMR analyses of complex 1

The complexation ratios of **1** prepared by methods 1 and 2 were determined from the integral areas in the solution ^{1}H NMR spectra, which were 1:2 in both methods.

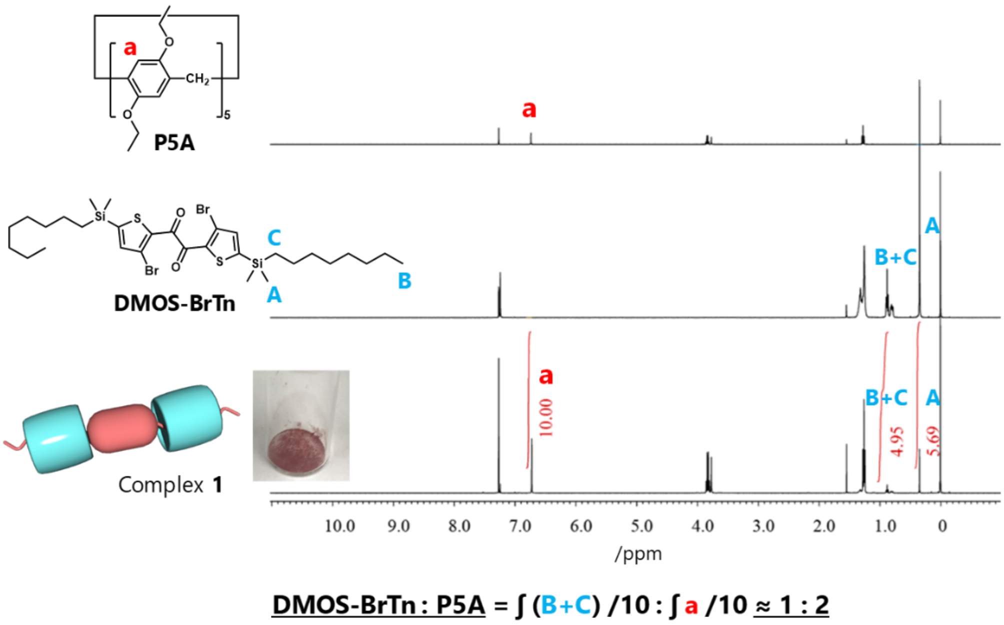

Figure S2. ^{1}H NMR spectra ($CDCl_3$, 25 °C) of **P5A** (top), **DMOS-BrTn** (middle), and complex **1** prepared by method 1 (bottom).

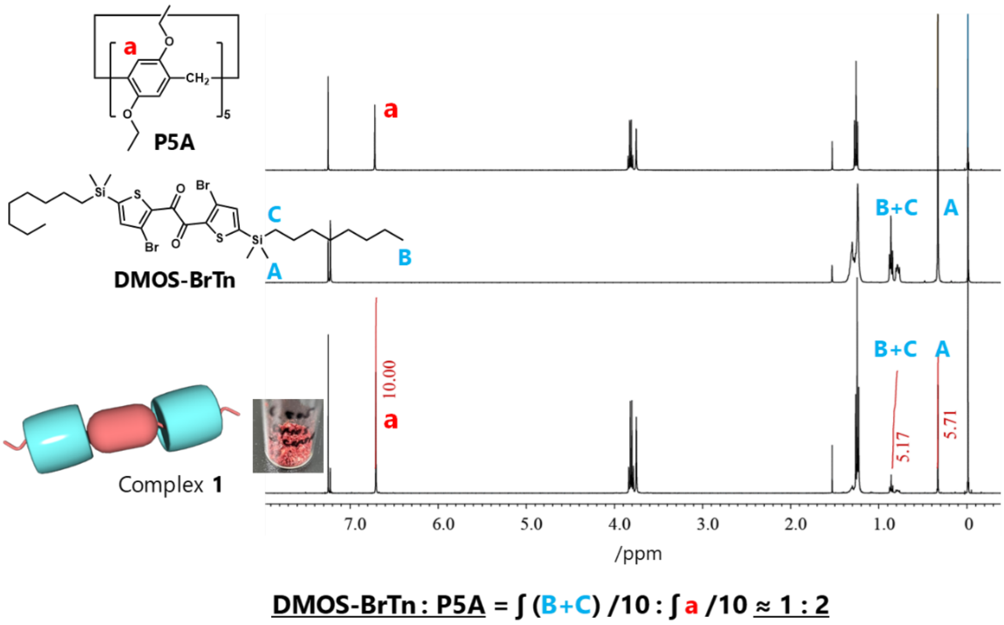

Figure S3. ^{1}H NMR spectra ($CDCl_3$, 25 °C) of **P5A** (top), **DMOS-BrTn** (middle), and complex **1** prepared by method 2 (bottom).

PXRD analyses of complex 1

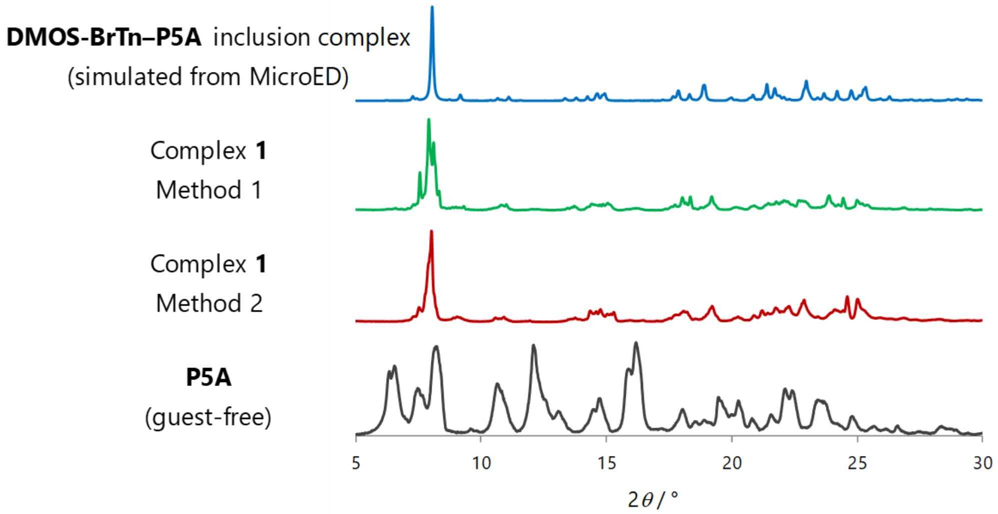

Figure S4. PXRD patterns of **DMOS-BrTn–P5A** inclusion complex (simulated from MicroED), complex **1** prepared by method 1, complex **1** prepared by method 2, and guest-free **P5A** (from top to bottom).

3. MicroED Data Collection and Processing

MicroED datasets of (**DMOS-BrTn**)·(**P5A**)$_2$ were measured on a Talos Arctica microscope (200 kV, parallel illumination, ~ 0.05 e/Å^2/sec) with SerialEM[2] as described in our previous reports.[3] Samples were loaded on a Quantifoil Mo R1.2/1.3 grid and kept at ~ 79 K. Diffraction patterns were recorded on a Ceta camera at the virtual camera distance of ~ 618 mm. The goniometer rotation speed corresponded to ~ 0.953°/frame. Crystals were plates or wedges (Figure S5a).

The datasets were processed in DIALS.[4] Data processing was accelerated by GNU parallel.[5] Of 40 measured crystals, 23 crystals were selected by xia2.multiplex[6] and scaled by dials.scale.[7] Only a single major cluster was observed in the scatter plot of the unit cell parameters (Figure S5b). The merging statistics are shown in Table S2. The structure was phased by SHELXT[8] and refined kinematically in SHELXL[9] under the Olex2 GUI.[10] All hydrogen atoms were placed on the calculated positions and refined using the riding model.

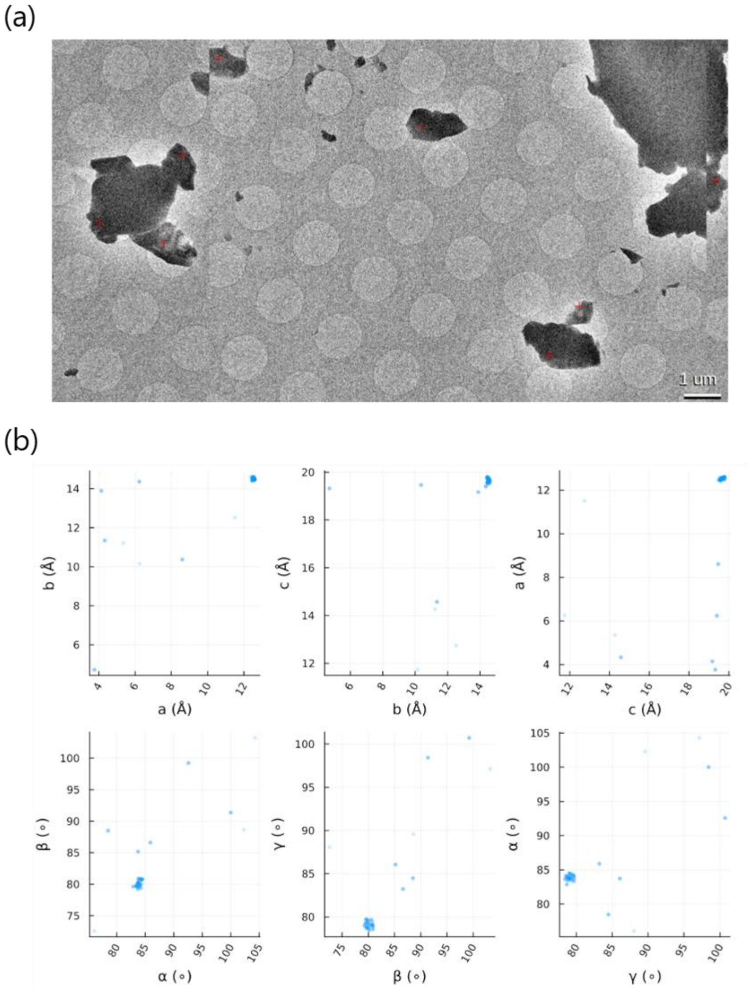

Figure S5. (a) Nanocrystals of (**DMOS-BrTn**)·(**P5A**)$_2$ observed on the MicroED grid. Red crosses indicate measurement positions. Image gaps are due to alignment errors in the SerialEM montaging process. (b) Unit cell distributions of (**DMOS-BrTn**)·(**P5A**)$_2$. Out of 40 measured crystals, 34 crystals better than 1.2 Å are shown. This is raw data before applying outlier rejection and contains low quality and/or mis-indexed crystals.

Table S2. MicroED Merging Statistics for (DMOS-BrTn)·(P5A)$_2$

d_max	d_min	#obs	#uniq	mult.	%comp	<I/sI>	r_pim	cc1/2
6.08	2.14	5384	679	7.93	96.72	35.3	0.038	0.982*
2.14	1.71	6867	699	9.82	100.00	20.4	0.052	0.991*
1.71	1.50	7366	702	10.49	100.00	13.7	0.069	0.985*
1.50	1.36	7428	706	10.52	100.00	9.5	0.083	0.973*
1.36	1.27	7444	699	10.65	100.00	9.1	0.082	0.969*
1.27	1.19	7525	704	10.69	100.00	7.9	0.082	0.977*
1.19	1.13	7519	692	10.87	100.00	6.9	0.084	0.974*
1.13	1.08	7488	695	10.77	100.00	5.8	0.097	0.958*
1.08	1.04	7558	687	11.00	100.00	4.5	0.113	0.941*
1.04	1.01	7770	728	10.67	100.00	3.2	0.144	0.919*
1.01	0.98	7513	683	11.00	100.00	2.6	0.170	0.875*
0.98	0.95	7715	702	10.99	100.00	2.1	0.193	0.813*
0.95	0.92	7824	719	10.88	100.00	1.9	0.221	0.747*
0.92	0.90	7495	674	11.12	100.00	1.6	0.252	0.712*
0.90	0.88	7775	718	10.83	100.00	1.1	0.320	0.666*
0.88	0.86	7475	682	10.96	100.00	1.0	0.374	0.567*
0.86	0.84	8122	723	11.23	100.00	0.9	0.406	0.585*
0.84	0.83	7365	678	10.86	100.00	0.8	0.444	0.526*
0.83	0.81	8001	727	11.01	100.00	0.7	0.523	0.388*
0.81	0.80	7458	666	11.20	100.00	0.7	0.561	0.399*
6.08	0.80	149092	13963	10.68	99.84	6.4	0.094	0.985*

Table S3. Crystallographic Data for Complex 1

Empirical formula	$C_{30}H_{48}Br_2O_2S_2Si_2$, 2($C_{55}H_{70}O_{10}$)
FW	2503.13
T / K	79
Crystal system	triclinic
Space group	P-1
a / Å	12.5208(9)
b / Å	14.4600(9)
c / Å	19.6291(16)
α / °	83.559(6)
β / °	80.254(7)
γ / °	79.200(6)
V / $Å^3$	3428.6(5)
Z	1
D (calcd) / g·cm^{-3}	1.212
GOF	1.186
R1 [$I > 2\sigma(I)$]*	0.1704
wR2 (all data)*	0.4086
High resolution limit for refinement / Å	0.80
COD ID.	3000627
CCDC No.	2504535

* We refined the atomic model only kinematically, ignoring contributions of multiple scattering (dynamical diffraction). This led to high R1 and wR2 values. Estimated standard deviations of the unit cell parameters and refined parameters are highly under-estimated and of no quantitative value.

4. Theoretical Calculations

All the theoretical calculations were performed using Gaussian 16 program package.[11] None of the optimized structures had imaginary frequencies.

Simulation of the absorption spectra

Single-point calculations were conducted using time-dependent density-functional theory (TDDFT) method at the CAM-B3LYP-D3/6-31G(d) level of theory.[12] All the geometries were extracted from the crystal structure. The natural transition orbitals for the S_0–S_1 transition were obtained at the same level of theory.

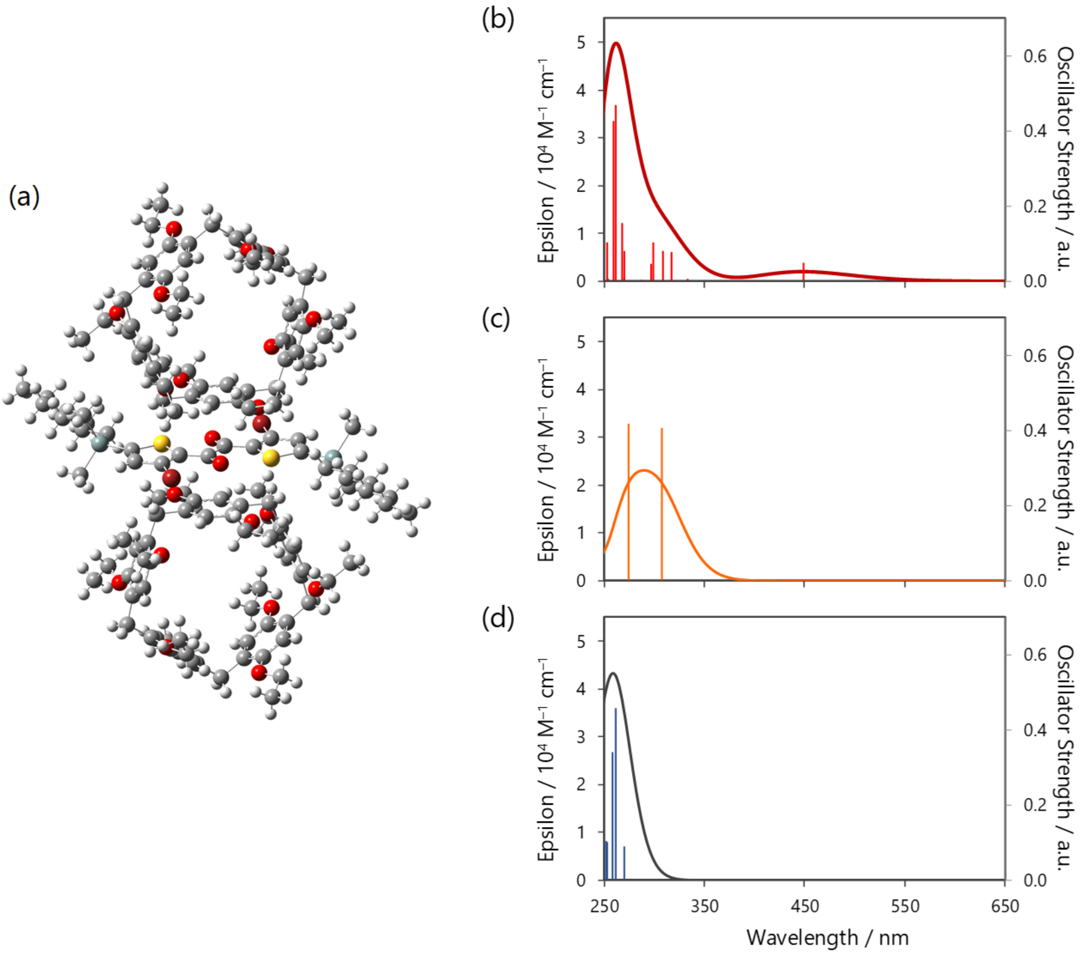

Figure S6. (a) Geometry of **DMOS-BrTn**–**P5A** 1:2 sandwich complex extracted from the crystal structure. (b–d) Simulated absorption spectra of (b) the complex, (c) **DMOS-BrTn**, and (d) **P5A** calculated at the CAM-B3LYP-D3/6-31G(d) level of theory. Bars represent the corresponding oscillator strength. In panel d, the intensities were doubled because there are two molecules of **P5A** in the complex.

Evaluation of the frontier orbital energies

Geometry optimizations and frequency calculations of **DMOS-BrTn** and permethoxy P5A were performed using DFT method at the B3LYP-D3/6-311G(d) level of theory.

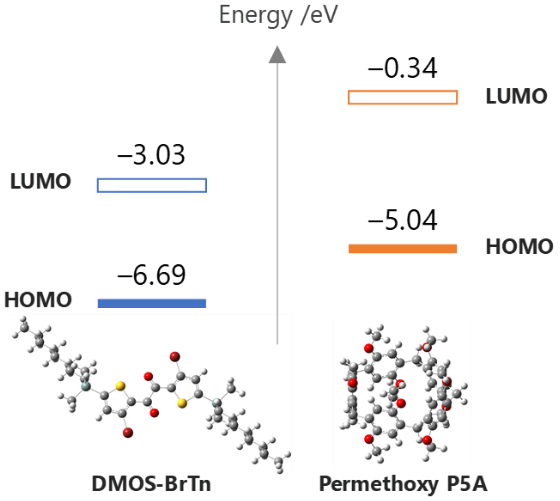

Figure S7. Energy diagram of the frontier molecular orbitals and optimized geometries for **DMOS-BrTn** and permethoxy P5A calculated at the B3LYP-D3/6-311G(d) level of theory.

Evaluation of the T_1 energy levels of DMOS-BrTn and 2,5-dimethoxytoluene P1

TDDFT calculations of **DMOS-BrTn** (optimized T_1-minimum geometry at UB3LYP-D3/6-311G(d)) and **P1** (optimized S_0-minimum geometry at B3LYP-D3/6-311G(d)) were performed using the Tamm–Dancoff approximation (TDA) to TDDFT method at the uCAM-B3LYP-D3/6-311G(d) level of theory.[13] The obtained T_1 energy levels were 2.11 and 3.67 eV for **DMOS-BrTn** and **P1**, respectively.

5. Photophysical Properties

Quenching experiment

PL spectra and decay curves of **DMOS-BrTn** in cyclohexane (spectral grade, 1.0×10^{-4} M) were acquired at RT in air, with varying amount of 2,5-dimethoxytoluene **P1**. Excitation wavelength was 368 nm, and the PL decay was recorded at 570 nm. Note that the decay curve with the largest amount of **P1** (the shortest lifetime) was acquired in a time-correlated single photon counting mode. The ratio of PL intensity at 568 nm (I_0/I) or the ratio of PL lifetime determined by single-exponential fits to the decay curves (τ_0/τ) was

plotted against the concentration of the quencher **P1** ([Q]). The plots can be fitted by the following Stern–Volmer equation:

$$I_0/I = 1 + k_{q,I} \cdot \tau_0 \cdot [Q]$$

$$\tau_0/\tau = 1 + k_{q,\tau} \cdot \tau_0 \cdot [Q]$$

where k_q is bimolecular quenching rate constant. The plots were well fitted to provide $k_{q,I} = 4.0 \times 10^8\ M^{-1}\ s^{-1}$ ($R^2 = 0.9998$) and $k_{q,\tau} = 3.7 \times 10^8\ M^{-1}\ s^{-1}$ ($R^2 = 0.9996$), indicating that **P1** quenches the triplet state of **DMOS-BrTn**.

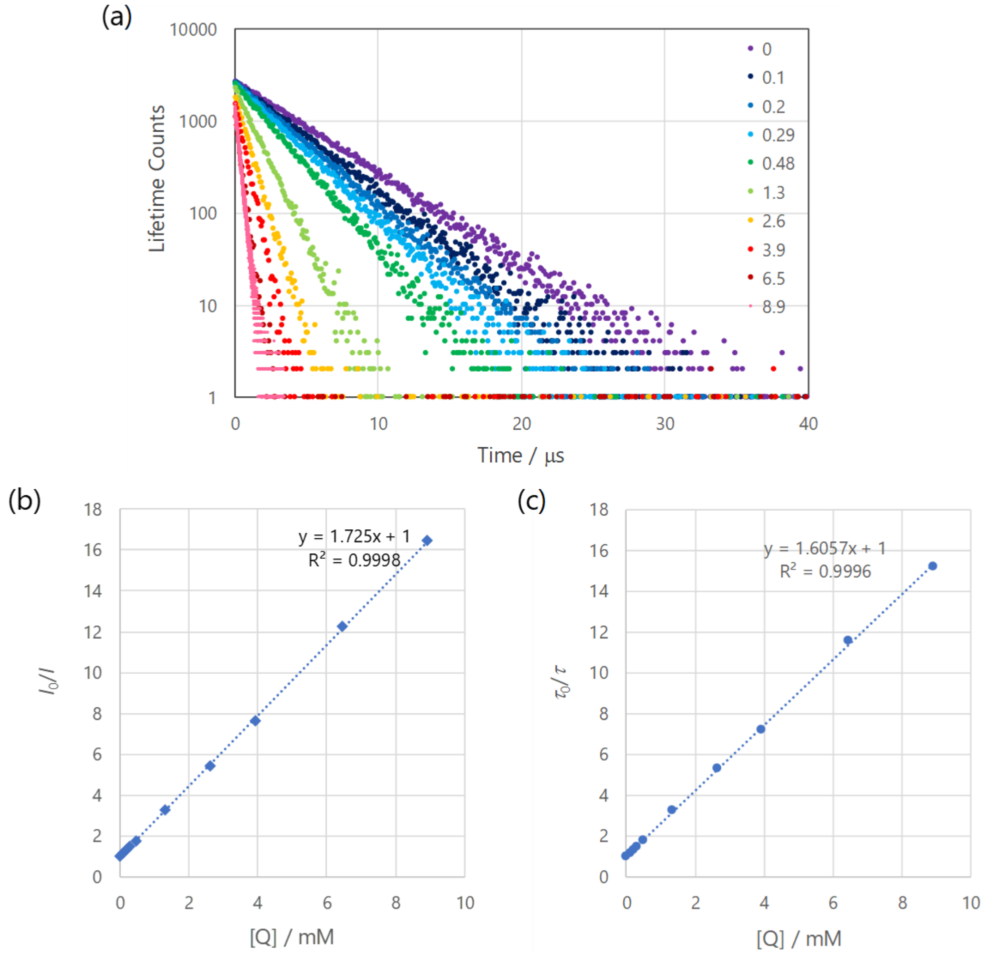

Figure S8. (a) PL decay curve of **DMOS-BrTn** in cyclohexane (1.0×10^{-4} M) at RT in air with varying concentration of **P1** (excited at 368 nm, detected at 570 nm). Values in the legend are [Q] / mM. (b, c) The Stern–Volmer plot on (b) I_0/I and (c) τ_0/τ.

Vapor response

Procedure for PL quantum yield measurement:

A small amount of complex **1** was placed in a quartz Petri dish (Hamamatsu photonics #A10095-03; Figures 2a and 3a left and Figure S9a). The PL quantum yield (PLQY) was measured, but no emission was observed at this stage (Figure S10, blue line). Then, this dish was placed in a glass container with cotton placed at the bottom. Approximately 15 drops of hexane were added to the cotton, and the glass container was covered with a lid (Figure S9b). The red color of the complex was gradually faded (Supporting Movie S1). After approximately 10 min (Figure S9c), the lid was removed, the Petri dish was taken out (Figure S9d), and the PLQY was measured (Figure S10, orange line). PLQY was determined as an average of four measurements to be 1.0% .

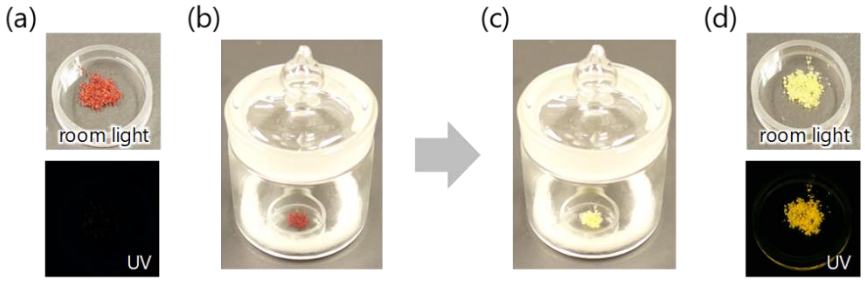

Figure S9. Photographic images of the hexane vapor response of complex **1**. (a) Initial state. (b,c) Set up of the vapor exposure: (b) just after starting the exposure; (c) after approximately 10 minutes. (d) After the exposure.

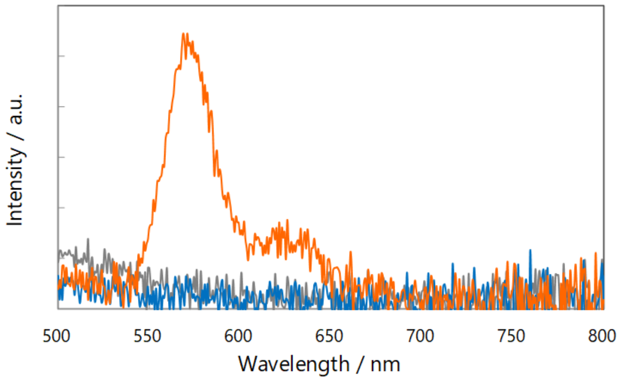

Figure S10. PL spectra acquired using a Hamamatsu photonics C9920-02 spectrometer with an integrating sphere (λ_{ex} = 368 nm). Grey line, reference (blank dish); blue line, complex **1** before hexane vapor exposure; orange line, after exposing to hexane vapor for approximately 10 minutes.

Procedure for the time course of PL spectra during hexane vapor exposure (Figure 3b):
A small amount of complex **1** was placed at the bottom of a quartz tube, and cotton was packed in the upper part of the tube. Several drops of hexane were added to the cotton, and the tube was sealed with a septum. PL spectra were acquired eleven times at 3-minute intervals, followed by a final measurement after 30 minutes (λ_{ex} = 368 nm, with a U340 band-pass filter and a L42 long-pass filter).

Procedure for PL lifetime measurement:
Sample of complex **1** after exposing to hexane vapor was prepared in the same way as for the quantum yield measurement. The area-weighted average PL lifetime was determined as 2.8±0.10 µs from a double-exponential fit to the decay curve using a HORIBA EzTime software.

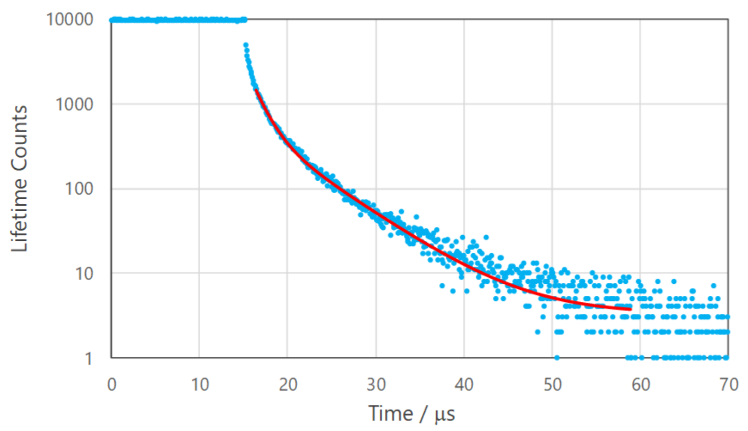

Figure S11. PL decay curve of complex **1** after exposing to hexane vapor, measured at RT in air (λ_{ex} = 368 nm). The red line is the double-exponential fit to the curve. PL intensity was recorded at 570 nm.

Procedure for excitation spectrum measurement:
Sample of complex **1** after exposing to hexane vapor was prepared in the same way as for the quantum yield measurement. Excitation spectrum was acquired (λ_{em} = 570 nm, with a L42 long-pass filter), which showed dip in the absorption region of **P5A** (Figure S12).

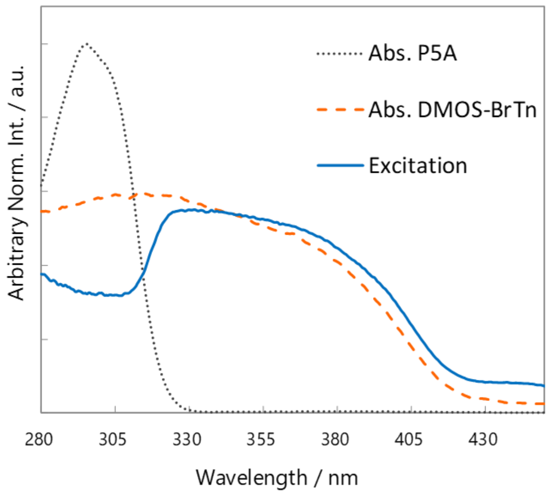

Figure S12. Excitation spectrum of complex **1** after exposing to hexane vapor (blue line, λ_{em} = 570 nm). Absorption spectra of solid **P5A** (black dotted line) and liquid **DMOS-BrTn** (orange broken line) are also shown for comparison.

Real-time observation of the vapor response under an optical and photoluminescence microscope

Borosilicate cover glasses (Matsunami Glass) were cleaned by sonication in a 20% detergent solution (Cleanace, AS ONE Corporation) for 4 h, followed by more than ten rinses with distilled water and ultrapure water (Milli-Q). A sample powder was dispersed onto the cleaned cover glass, which was then placed in a sample chamber together with a piece of filter paper. Immediately after initiating microscopy imaging, a drop of *n*-hexane was added to the filter paper to monitor changes in the crystal structure and emission. The chamber was then sealed with a glass plate to minimize solvent vapor loss.

Optical microscopy measurements were performed using a stereo microscope (SMZ800N, Nikon) equipped with a CMOS color camera (DS-Fi3, Nikon) at 50 ms per frame. Photoluminescence microscopy measurements were carried out using an inverted fluorescence microscope (Ti-E, Nikon). A 405 nm continuous-wave (CW) laser (OBIS 405LX, Coherent; 68 μW) was used for sample excitation through an oil-immersion objective lens (CFI Plan Apo λ 100× Oil, NA 1.45, Nikon).[14] The same objective lens was used to collect the emission from the particles. A dichroic mirror (Di02-R405, Semrock) and a long-pass filter (ET425lp, Chroma) were used to suppress scattered excitation light. Color emission images were acquired with a scientific CMOS color camera (Dhyana 400DC, Tucsen Photonics) at 500 ms per frame. All measurements were conducted at room temperature.

Vapor Shape Selectivity

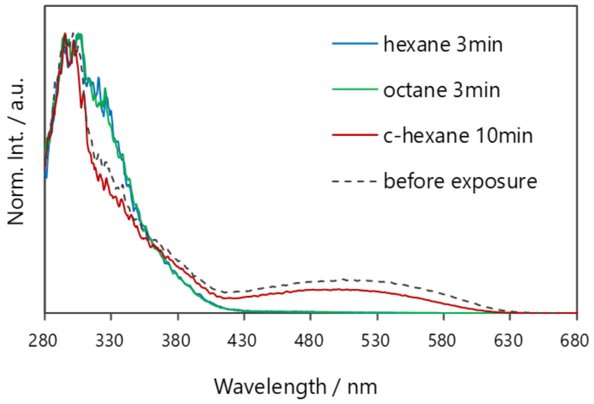

Figure S13. Absorption spectra (Kubelka–Munk conversion of diffuse reflectance spectra) of complex **1** before (black broken line) and after exposing to: hexane vapor for 3 min (blue line), octane vapor for 3 min (green line), and cyclohexane vapor for 10 min (red line).

Microscopic observation of the vapor-induced phase separation

Photographic images under microscope presented in Figure 4b were taken using a SONY NEX-5N camera attached to the OLYMPUS BX60M fluorescence microscope with a MICRONET NY-1S adaptor. An OLYMPUS mirror unit (U-MBFL3, longpass, $\lambda > 400$ nm, < 10 mW cm^{-2}) was used for recording the bright-field (BF) and polarizing images (POM) while a U-MWUS ($\lambda_{max} = 365$ nm) unit was used for the PL image (LM).

Supporting Movie 1. Complex **1** during *n*-hexane vapor exposure in a glass container.

Supporting Movie 2. Optical microscopy observation of complex **1** during *n*-hexane vapor exposure (5x speed, whole region: 1570 μm × 1116 μm).

Supporting Movie 3. Optical microscopy observation of complex **1** during *n*-hexane vapor exposure (5x speed, magnified view: 100 μm × 100 μm).

Supporting Movie 4. Optical microscopy observation of complex **1** during *n*-hexane vapor exposure (5x speed, magnified view: 130 μm × 130 μm).

Supporting Movie 5. Optical microscopy observation of complex **1** during *n*-hexane vapor exposure (5x speed, magnified view: 140 μm × 140 μm).

Supporting Movie 6. Luminescence microscopy observation of complex **1** during *n*-hexane vapor exposure (5x speed, 34.667 μm × 34.667 μm).

6. References

1. (a) Y. Tani, M. Terasaki, M. Komura and T. Ogawa, *J. Mater. Chem. C*, 2019, **7**, 11926-11931; (b) T. Ogoshi, K. Kitajima, T. Aoki, S. Fujinami, T.-a. Yamagishi and Y. Nakamoto, *J. Org. Chem.*, 2010, **75**, 3268-3273.
2. D. N. Mastronarde, *Microsc. Microanal.*, 2003, **9**, 1182-1183.
3. (a) D. Gogoi, T. Sasaki, T. Nakane, A. Kawamoto, H. Hojo, G. Kurisu and R. Thakuria, *Cryst. Growth Des.*, 2023, **23**, 5821-5826; (b) T. Sasaki, T. Nakane, A. Kawamoto, T. Nishizawa and G. Kurisu, *CrystEngComm*, 2023, **25**, 352-356.
4. (a) M. T. B. Clabbers, T. Gruene, J. M. Parkhurst, J. P. Abrahams and D. G. Waterman, *Acta Crystallographica Section D*, 2018, **74**, 506-518; (b) G. Winter, D. G. Waterman, J. M. Parkhurst, A. S. Brewster, R. J. Gildea, M. Gerstel, L. Fuentes-Montero, M. Vollmar, T. Michels-Clark, I. D. Young, N. K. Sauter and G. Evans, *Acta Crystallographica Section D*, 2018, **74**, 85-97.
5. O. Tange, *Journal*, 2011, **36**, 42-47.
6. R. J. Gildea, J. Beilsten-Edmands, D. Axford, S. Horrell, P. Aller, J. Sandy, J. Sanchez-Weatherby, C. D. Owen, P. Lukacik, C. Strain-Damerell, R. L. Owen, M. A. Walsh and G. Winter, *Acta Crystallographica Section D*, 2022, **78**, 752-769.
7. J. Beilsten-Edmands, G. Winter, R. Gildea, J. Parkhurst, D. Waterman and G. Evans, *Acta Crystallographica Section D*, 2020, **76**, 385-399.
8. G. Sheldrick, *Acta Crystallographica Section A*, 2015, **71**, 3-8.
9. G. Sheldrick, *Acta Crystallographica Section C*, 2015, **71**, 3-8.
10. O. V. Dolomanov, L. J. Bourhis, R. J. Gildea, J. A. K. Howard and H. Puschmann, *J. Appl. Crystallogr.*, 2009, **42**, 339-341.
11. M. J. Frisch, G. W. Trucks, H. B. Schlegel, G. E. Scuseria, M. A. Robb, J. R. Cheeseman, G. Scalmani, V. Barone, G. A. Petersson, H. Nakatsuji, X. Li, M. Caricato, A. V. Marenich, J. Bloino, B. G. Janesko, R. Gomperts, B. Mennucci, H. P. Hratchian, J. V. Ortiz, A. F. Izmaylov, J. L. Sonnenberg, D. Williams-Young, F. Ding, F. Lipparini, F. Egidi, J. Goings, B. Peng, A. Petrone, T. Henderson, D. Ranasinghe, V. G. Zakrzewski, J. Gao, N. Rega, G. Zheng, W. Liang, M. Hada, M. Ehara, K. Toyota, R. Fukuda, J. Hasegawa, M. Ishida, T. Nakajima, Y. Honda, O. Kitao, H. Nakai, T. Vreven, K. Throssell, J. A. Montgomery Jr., J. E. Peralta, F. Ogliaro, M. J. Bearpark, J. J. Heyd, E. N. Brothers, K. N. Kudin, V. N. Staroverov, T. A. Keith, R. Kobayashi, J. Normand, K. Raghavachari, A. P. Rendell, J. C. Burant, S. S. Iyengar, J. Tomasi, M. Cossi, J. M. Millam, M. Klene, C. Adamo, R. Cammi, J. W. Ochterski, R. L. Martin, K. Morokuma, O. Farkas, J. B. Foresman and D. J. Fox, *Gaussian 16 Rev. C.01*, Wallingford, CT, 2019.

12. (a) T. Yanai, D. P. Tew and N. C. Handy, *Chem. Phys. Lett.*, 2004, **393**, 51-57; (b) S. Grimme, J. Antony, S. Ehrlich and H. Krieg, *J. Chem. Phys.*, 2010, **132**.
13. (a) M. J. G. Peach and D. J. Tozer, *J. Phys. Chem. A*, 2012, **116**, 9783-9789; (b) I. Tamm, *J. Phys. USSR*, 1945, **9**, 449-460; (c) S. M. Dancoff, *Phys. Rev.*, 1950, **78**, 382-385; (d) S. Hirata and M. Head-Gordon, *Chem. Phys. Lett.*, 1999, **314**, 291-299; (e) M. E. Casida, F. Gutierrez, J. Guan, F.-X. Gadea, D. Salahub and J.-P. Daudey, *J. Chem. Phys.*, 2000, **113**, 7062-7071.
14. M. Yamashita, S. Nagai, S. Ito and T. Tachikawa, *J. Phys. Chem. Lett.*, 2021, **12**, 7826-7831.
